# Guided simulation of conditioned chemical reaction networks

**DOI:** 10.1007/s11203-025-09326-9

**Published:** 2025-05-17

**Authors:** Marc Corstanje, Frank van der Meulen

**Affiliations:** Department of Mathematics, Vrije Universiteit Amsterdam, Amsterdam, The Netherlands

**Keywords:** Chemical reaction processes, Doob’s *h*-transform, Exponential change of measure, Guided process, 60J27, 60J28, 60J74

## Abstract

Let *X* be a chemical reaction process, modeled as a multi-dimensional continuous-time jump process. Assume that at given times $0< t_1< \cdots <t_n$, linear combinations $v_i = L_i X(t_i),\, i=1,\dots ,n$ are observed for given matrices $L_i$. We show how the process that is conditioned on hitting the states $v_1,\dots , v_n$ is obtained by a change of measure on the law of the unconditioned process. This results in an algorithm for obtaining weighted samples from the conditioned process. Our results are illustrated by numerical simulations.

## Introduction

Chemical reaction networks are used to study a wide class of biological, physical and chemical processes that evolve over time. For instance, one can think of the transcription of genes to mRNA and then the translation to protein, the kinetics of a virus or the dynamics of chemical components reacting with each other. The forward evolution of such processes can be described in different ways: *(i)* a system of ordinary differential equations, see e.g. Feinberg (2019), *(ii)* a system of stochastic differential equations, see e.g. Fuchs (2013) or *(iii)* continuous-time Markov jump processes, as in Anderson (2007). It is the third option that we consider in this paper.

### Chemical reaction networks

Chemical reactions are described as linear combinations of chemical components merging into each other. Typically, one denotes a reaction in which components *A* and *B* are merged into *C* and *D* by

1.1 $$\begin{aligned} A+B \rightarrow C + D.\end{aligned}$$

More generally, a *chemical reaction network* consists of:

- a *Species* set $\mathcal {S} = \left\{ S_1, \dots , S_d\right\}$ which consists of the chemical components whose counts we model;
- a *Reaction* set $\mathcal {R}$. A reaction $\ell \in \mathcal {R}$ is modeled as $\sum _{k}\nu _{k\ell }S_k\rightarrow \sum _k \nu _{k\ell }' S_k$. We characterize $\ell$ by the change of counts of the species $\xi _\ell = \left( \nu _{k\ell }'-\nu _{k\ell }\right) _{k=1}^d$.

For example, in (1.1), the species set is $\{A,B,C,D\}$ and $\xi$ is given by $(-1,-1,1,1)$. Let ${\mathbb S}\subseteq {\mathbb Z}_{\ge 0}^d$ denote the state space of the reaction system. A vector in ${\mathbb S}$ is of the form $(x_k)_{k=1}^d$, where $x_k$ denotes the species count of type $S_k$. We study a Markov process $X = \left( X(t)\right) _{t\ge 0}$ on ${\mathbb S}$ that models the evolution of species counts over time. That is, at time *t*, the *k*-th element of *X*(*t*) is given by $\# S_k(t)$. We assume that the initial state $X(0)=x_0$ of the process is known. At a given time *t*, the time until a reaction of type $\ell$ takes place is given by $\tau _\ell$. When reaction $\hat{\ell } = \mathop {\text {argmin}}\limits _{\ell \in \mathcal {R}}\tau _\ell$ occurs, the process jumps at time $t+\tau _{\hat{\ell }}$ to $X\left( t+\tau _{\hat{\ell }}\right) = X(t)+\xi _{\hat{\ell }}$. The reactions are assumed to occur according to an inhomogeneous Poisson process with intensity function that we refer to as the *reaction rate*. A more detailed description of the stochastic model is given in Sect. 2. The process *X* evolving on the chemical reaction network is referred to as the *chemical reaction process*.

### Statistical problem

Suppose at fixed times $0<t_1<\cdots <t_n$, we observe $v_1,\dots v_n$, where $v_k=L_k X(t_k)$ with $L_k\in {\mathbb R}^{m_k\times d}$ and $m_k \le d$, $k=1,\dots n$. Not assuming *L* to be the identity matrix is for example important in applications where the measuring device cannot distinguish two or more species, so that only sums of their counts are observed. We will assume the rows of each $L_k$ to be linearly independent. Typically reaction rates are unknown and we wish to infer those from the data. Suppose the reaction rate depend on a parameter vector $\theta$. Likelihood-based inference for $\theta$ is hampered by the lack of closed-form expressions for the transition probabilities of *X*. However, if the process were observed continuously over time, the problem would be easier. Therefore, it is natural to employ a data-augmentation scheme where we iteratively sample *X* on $[0,t_n]$ conditional on $v_1,\dots , v_n$ and $\theta$ and then update $\theta$ conditional on *X*. In this paper, we focus on the first step, sampling from $\left( X \mid L_k X(t_k)=v_k,\, k=1,\dots n\right)$. It is a key objective of this paper to show rigorously how this can be done efficiently. Note that a simple rejection sampling scheme where we discard all paths contradicting the observations is valid but very inefficient in most settings.

### Approach: conditioning by guiding

Our approach builds on earlier work in Corstanje et al. (2023) for general Markov processes in case of a single observation. Let us highlight the main points. The law of the process *X*, conditioned to be in a given state at fixed times, is obtained through Doob’s *h*-transform. That is, there is a function $h:[0,T]\times {\mathbb S}\rightarrow \mathbb {R}_+$ that depends on the transition probabilities of *X* which induces a change of measure. Under the new measure, ${\mathbb P}^{h}$, the process is conditioned to hit the observed states at times of observation. Since *h* is typically unknown, we replace it by a fully tractable function $g:[0,T]\times {\mathbb S}\rightarrow \mathbb {R}_+$ that itself induces a change of measure to a measure ${\mathbb P}^{g}$. Under certain conditions, ${\mathbb P}^{h}$ is absolutely continuous with respect to ${\mathbb P}^{g}$ and

1.2 $$\begin{aligned} {\frac{\,\textrm{d}{\mathbb P}^{h}}{\,\textrm{d}{\mathbb P}^{g}}}(X)=\frac{1}{h(0,x_0)}F(X). \end{aligned}$$

Here, *F* is known in closed form, depends on *g* but does not depend on *h*. Weighted samples of the conditioned process can therefore be obtained by sampling under ${\mathbb P}^g$. The argument for making the above precise is not too hard in the case where *g* is bounded and bounded away from zero. However, some natural choices we discuss and have been proposed in the literature require a more delicate argument.

### Related literature

Statistical inference for chemical reaction processes has received considerable attention over the past decade. In this section we summarise related work, while in the next section we highlight contributions of this paper. Rathinam and Yu (2021) consider the setting where one observes a subset of the species counts *continuously* over time and wants to filter the latent species counts. Reeves and Bhat (2022) parametrise model transition rates by neural networks, while assuming all trajectories are *fully continuously* observed. Parameter estimation is then done by gradient ascent to maximise the log likelihood.

In this paper, we consider *discrete-time* observations and therefore the works below are more closely related to our work. Warne et al. (2019) give an introduction to chemical reaction processes and consider estimation for discrete-time partial observations with Gaussian noise focussing on Approximate Bayesian Computation. Fearnhead (2008) and Golightly and Sherlock (2019) are probably closest related to our approach. The common starting points of these works is a slightly informal computation that reveals how the reaction rate of the chemical reaction processes changes upon conditioning the process on a future observation (we present this argument at the start of Sect. 3). While the reaction rate for the conditioned process is intractable, it can be approximated and this simply boils down to choosing *g* as above. Fearnhead (2008) approximates *g* using Euler discretisation of the Chemical Langevin Equation (CLE) assuming the process is fully observed without error. Golightly and Sherlock (2019) consider conditioning on a partial observation corrupted by Gaussian noise. Their choice of *g* is based on the linear noise approximation to the CLE. This is shown to outperform the approach of Fearnhead (2008) and earlier work in Golightly and Wilkinson (2015).

Georgoulas et al. (2017) construct an unbiased estimator for the likelihood using random truncations and computation of matrix exponentials. This in turn is used to exploit the pseudo-marginal MCMC algorithm (Andrieu and Roberts 2009) for parameter estimation. Building upon this work Sherlock and Golightly (2023) introduce the minimal extended statespace algorithm and the nearly minimal extended statespace algorithm to alleviate the problem of choosing a proposal distribution for the truncation level, as required in Georgoulas et al. (2017).

Alt and Koeppl (2023) consider the same setting as we do and derive approximations to the filtering and smoothing distributions using expectation propagation.

### Contribution

We provide sufficient conditions on *g* such that (1.2) holds true. We extend the result in Corstanje et al. (2023) for a single complete observation to multiple partial observations in the context of chemical reaction processes. Moreover, we discuss a variation of the *next reaction* algorithm by Gillespie (1976) for sampling from a class of reaction networks with unbounded time-dependent reaction intensities.

The proposed methods fit within the framework of Backward Filtering Forward Guiding (van der Meulen and Schauer 2020), drawing strongly on techniques for exponential changes of measure as outlined in Palmowski and Rolski (2002). This enables us to construct a *guided* process that at any time takes into account *all* future conditionings.

Compared to Fearnhead (2008) and Golightly and Sherlock (2019), we consider the setting of multiple future conditionings (rather than one), without imposing extrinsic noise on the observations. Moreover, we derive the conditioned process and likelihood ratio in (1.2) on path space. Sufficient conditions on *g* to guarantee absolute continuity are given in Theorem 3.4. It turns out that the choices for *g* in Fearnhead (2008) and Golightly and Sherlock (2019) satisfy the assumptions of this theorem. In numerical examples we show that flexibility in choosing *g* is particularly beneficial in cases where some of the components of the chemical reaction process have counts that vary monotonically over time.

### Outline

We introduce stochastic chemical reaction processes in Sect. 2 and discuss examples that we will study. In Sect. 3, we describe the approach of conditioning chemical reaction processes by guiding and present conditions on *g* such that ${\mathbb P}^h$ is absolutely continuous with respect to ${\mathbb P}^g$. In Sect. 4 we consider various choices for *g*. Conditions for equivalence of ${\mathbb P}^h$ and ${\mathbb P}^g$ are discussed in Sect. 5. In Sect. 6 we present methods for simulation of conditioned chemical reaction processes together with numerical illustrations. We end with a discussion section. The appendix contains various proofs.

### Frequently used notation

Throughout, we assume that we have an underlying probability space $\left( \Omega , \mathcal {F}, {\mathbb P}\right)$. For a stochastic process *X*, we use the notation $X^t = \{ X(s) :s\le t\}$. Given $L \in {\mathbb R}^{m\times d}$ with $m\le d$ and $v\in {\mathbb R}^m$, we denote the inverse image of *v* under *L* by $L^{-1}v = \{x\in {\mathbb R}^d: Lx=v\}$. For functions $f_1(t,x)$ and $f_2(t,x)$ of time and space, we say that $f_1\propto f_2$ if there exists a differentiable function $\kappa$ of time such that for all *t*, *x*, $f_1(t,x) = \kappa (t)f_2(t,x)$. Derivatives with respect to a variable representing *t*, say $\partial / \partial t$ are denoted by $\partial _t$. We denote by

1.3 $$\begin{aligned} A_n=\{ L_kX(t_k)=v_k,\, k=1,\dots ,n \}, \end{aligned}$$

a set of conditionings. If a measure $\mu$ is absolutely continuous with respect to $\nu$, we write $\mu \ll \nu$.

## Chemical reaction processes

We construct a stochastic process to model the dynamics of the chemical reaction network described in Sect. 1.1 following Chapter 1 of Anderson and Kurtz (2015). Let *X* be a Markov process on ${\mathbb S}$ such that the *i*-th component of *X*(*t*), $X_i(t)$, represents the frequency of species $S_i$ at time *t*. A reaction $\ell$ is represented through a difference vector $\xi _\ell \in {\mathbb S}$ and an intensity $\lambda _\ell :[0,\infty )\times {\mathbb S}\rightarrow [0,\infty )$. We assume

2.1 $$\begin{aligned} {\mathbb P}\left( X({t+\Delta })-X(t) = \xi _\ell \mid \mathcal {F}_t^X\right) = \lambda _\ell (t, X(t))\Delta + o(\Delta ), \qquad \Delta \downarrow 0, \end{aligned}$$

where $\mathcal {F}_t^X = \sigma (X^t)$. The jump probabilities specified in (2.1) correspond to a process with jumps $(\xi _\ell )_{\ell \in \mathcal {R}}$ and jump rate functions $(\lambda _\ell )_{\ell \in \mathcal {R}}$. Throughout, we impose the following assumptions on the network.

### Assumption 2.1

$(\lambda _\ell )_{\ell \in \mathcal {R}}$ and $(\xi _\ell )_{\ell \in \mathcal {R}}$ are such that

- $\lambda _\ell (t, x)\ge 0$ for all $t\ge 0$, $x\in {\mathbb S}$ and $\ell \in \mathcal {R}$.
- For all $\ell \in \mathcal {R}$, $\xi _\ell \in {\mathbb Z}^d$ is such $\lambda _\ell (t,x)>0$ implies $x+\xi _\ell \in {\mathbb S}$ for all $t\ge 0$ and $x\in {\mathbb S}$.
- For all $t\ge 0$ and $x\in {\mathbb S}$, $\int _0^t\sum _{\ell \in \mathcal {R}}\lambda _\ell (s,x) \,\textrm{d}s <\infty$.

For a stochastic process *X*(*t*), let $T_K=\inf \{ t: |X(t)|\ge K\}$ and $T_\infty =\lim _{K\rightarrow \infty } T_K$. Let *X* be the jump process with jumps $(\xi _\ell )_{\ell \in \mathcal {R}}$ and jump rate functions $(\lambda _\ell )_{\ell \in \mathcal {R}}$ satisfying

2.2 $$\begin{aligned} X(t) = x_0 + \sum _{\ell \in \mathcal {R}} \xi _\ell Y_\ell \left( \int _0^t \lambda _\ell (s, X(s))\,\textrm{d}s \right) , \qquad 0\le t< T_{\infty }, \end{aligned}$$

where $(Y_\ell )_{\ell \in \mathcal {R}}$ are independent, unit rate Poisson processes. We assume the process to be non-explosive: $\mathbb {P}(T_\infty <\infty )=0$. For $f:[0,T] \times {\mathbb S} \rightarrow {\mathbb R}$ with $f(t,\cdot )$ finitely supported for every *t* define

2.3 $$\begin{aligned} (\mathcal {L}f)(t,x) = \sum _{\ell \in \mathcal {R}} \lambda _\ell (t, x) \left[ f(t,x+\xi _\ell )-f(t,x)\right] , \end{aligned}$$

By Theorem 1.22 in Anderson and Kurtz (2015), there exists a filtration $(\mathcal {F}_t)_{t\ge 0}$ such that for all such *f*

$$\begin{aligned} D^f(t):=f(t, X(t))-\int _0^t (\partial _s + \mathcal {L}) f(s, X(s))\,\textrm{d}s \end{aligned}$$

is an $(\mathcal {F}_t)$-martingale. That is, *X* is the unique solution to the martingale problem for $\mathcal {A}:= \partial _t + \mathcal {L}$.

### Distribution of reaction times

To the $\ell$-th reaction, we associate a reaction time $\tau _\ell$, with distribution specified by

2.4 $$\begin{aligned} {\mathbb P}\left( \tau _\ell> \Delta \mid X(t)=x\right) = \exp \left( -\int _t^{t+\Delta } \lambda _\ell (s,x)\,\textrm{d}s\right) , \qquad t\ge 0, \Delta >0, x\in {\mathbb S}. \end{aligned}$$

If $\lambda _\ell$ is constant in time, it follows from (2.4) that $\tau _\ell \mid X(s)=x\sim \textrm{Exp}\left( \lambda _\ell (x)\right)$. This implies that the time $\tau =\min _{\ell \in \mathcal {R}}\tau _\ell$ that the first reaction occurs satisfies $\tau \mid X(s)=x \sim \textrm{Exp}\left( \sum _{\ell \in \mathcal {R}} \lambda _\ell (x)\right)$.

If the reaction times are inhomogeneous in time and Assumption 2.1 is satisfied, similarly the time for the first reaction to occur has distribution (2.4) with intensity function $\sum _{\ell \in \mathcal {R}}\lambda _\ell$. Moreover, the probability distribution of the first reaction in any subset $R\subseteq \mathcal {R}$ also satisfies (2.4) with intensity function $\sum _{\ell \in R}\lambda _\ell$.

### Chemical master equation and chemical Langevin equation

Denote the transition probabilities of (2.2) by *p*. That is $p(s,x;t,y) = {\mathbb P}\left( X(t)=y\mid X(s)=x\right)$. The Kolmogorov forward equation yields the *chemical master equation* given by

$$\begin{aligned} \partial _t p(s,x;t,y) = \sum _{\ell \in \mathcal {R}}\lambda _\ell (t,y-\xi _\ell )p(s,x;t,y-\xi _\ell ) - \sum _{\ell \in \mathcal {R}} \lambda _\ell (t,y)p(s,x;t,y), \end{aligned}$$

with initial condition $p(s,x;s,y)=1\{y=x\}$. It is well-known, see e.g. Li (2020) that the chemical reaction process can be approximated by solutions to the *Chemical Langevin Equation (CLE)*, which is the SDE

2.5 $$\begin{aligned} \,\textrm{d}Y(t) = b_{\textrm{CLE}}(t,Y(t)) \,\textrm{d}t + \sigma _{\textrm{CLE}}(t,Y(t)) \,\textrm{d}W_\ell (t),\qquad Y(0)=x_0 \end{aligned}$$

where (assuming the reactions to be numbered $1,\ldots ,{B}$)

2.6 $$\begin{aligned} \begin{aligned} b_{\textrm{CLE}}(t,x)&= \sum _{\ell =1}^{B} \lambda _\ell (t,x)\xi _\ell \\ \sigma _{\textrm{CLE}}(t,x)&= \begin{bmatrix} \xi _1&\ldots&\xi _{B} \end{bmatrix} \sqrt{\text{ diag }(\lambda _1(t,x),\ldots , \lambda _{{B}}(t,x))} \end{aligned} \end{aligned}$$

and *W* is an independent ${\mathbb R}^{B}$-valued Brownian motion.

### Examples

#### Example 2.2

(Pure death process) Our simplest example models a population of initial size $x_0$ in which an individual dies in a time interval $(t,t+\Delta )$ with probability $c\Delta + o(\Delta )$ for some constant $c>0$ and $\Delta$ small. Such a process is modelled as chemical reaction process with just one specie and one reaction, namely $\lambda _1:(t,x)\mapsto cx$ with $\xi _1 = -1$.

#### Example 2.3

(Gene Transcription and Translation *(GTT)*) A stochastic model for the process in which information is encoded in DNA and transferred to mRNA is described in Section 2.1.1 of Anderson and Kurtz (2015). The basic model considers the three species *Gene (G)*, *mRNA (M)* and *Protein (P)* in the set $\mathcal {S} = \{ G, M, P\}$. We consider four reactions.

1. **Transcription: **$G\rightarrow G+M$ with rate constant $\kappa _1$.
2. **Translation: **$M\rightarrow M+P$ with rate constant $\kappa _2>0$.
3. **Degradation of mRNA: **$M\rightarrow \emptyset$ with rate constant $d_M>0$.
4. **Degradation of protein: **$P\rightarrow \emptyset$ with rate constant $d_P>0$.

Let *X*(*t*) be the count vector at time *t* of species counts (*G*, *M*, *P*). In this example $(2.2)$ translates to

**Fig. 1:**
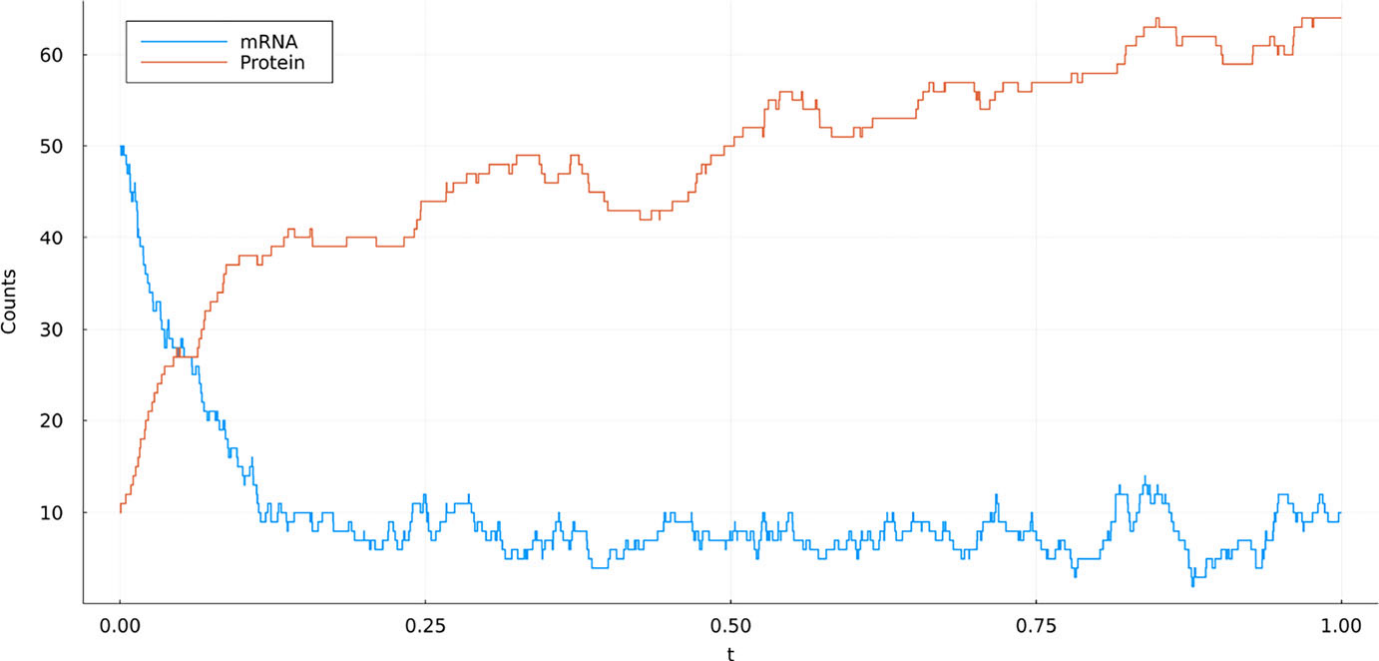
Realization of (2.7) using $\kappa _1 = 200$, $\kappa _2 = 10$, $d_M = 25$, $d_P = 1$ and initial position $x_0 = (1, 50, 10)$. Note that the gene count is constant in this process. Therefore it was omitted from the figure

2.7 $$\begin{aligned} \begin{aligned} X(t)&= x_0 + Y_1\left( \int _0^t \kappa _1 X_{1}(s)\,\textrm{d}s\right) \begin{pmatrix} 0 \\ 1 \\ 0 \end{pmatrix} + Y_2\left( \int _0^t \kappa _2 X_{2}(s)\,\textrm{d}s\right) \begin{pmatrix} 0 \\ 0 \\ 1 \end{pmatrix} \\&\quad + Y_3\left( \int _0^t d_M X_{2}(s)\,\textrm{d}s\right) \begin{pmatrix} 0 \\ -1 \\ 0 \end{pmatrix} + Y_4\left( \int _0^t d_P X_{3}(s)\,\textrm{d}s\right) \begin{pmatrix} 0 \\ 0 \\ -1 \end{pmatrix} \end{aligned}, \end{aligned}$$

where $Y_1,Y_2,Y_3,Y_4$ are independent unit rate Poisson processes. A realization of this process can be found in Fig. 1.

#### Example 2.4

(Enzyme kinetics) The standard model for describing enzyme kinetics, see e.g. Bersani et al. (2008), where a substrate binds an enzyme reversibly to form an enzyme-substrate complex, which can in turn deteriorate into an enzyme and a product. We thus model *Substrate (S)*, *Enzyme (E)*, *Enzyme-substrate (SE)* and *Product (P)* in the species set $\mathcal {S} = \left\{ S, E, SE, P\right\}$ and consider of the following reactions.

2.8 $$\begin{aligned} S+E \overset{\kappa _1}{\underset{\kappa _2}{\rightleftharpoons }} SE \overset{\kappa _3}{\longrightarrow }P+E \end{aligned}$$

Equivalently:

1. $S+E \rightarrow SE$ with rate constant $\kappa _1$.
2. $SE \rightarrow S+E$ with rate constant $\kappa _2$.
3. $SE\rightarrow P+E$ with rate constant $\kappa _3$.

Then the reaction rates corresponding to the above listed three reactions are given by

1. $$\lambda _1(x)=\kappa _1 x_1x_2$$ and $$\xi _1 = (-1,-1,1,0)$$.
2. $$\lambda _2(x) = \kappa _2 x_3$$ and $$\xi _2 = (1,1,-1,0)$$.
3. $$\lambda _3(x) = \kappa _3 x_3$$ and $$\xi _3 = (0,1,-1,1)$$.

Here $(x_1, x_2, x_3, x_4)$ refer to species counts of (*S*, *E*, *SE*, *P*). Interesting aspects of this example are firstly that the fourth component (*P*) only appears in reaction (3) where 1 is added and therefore is monotonically increasing and secondly that there are absorbing states such as $x= (0, x_2, 0, x_4)$ where the process is killed as all reaction rates are zero. A realization of this process can be found in Fig. 1.

**Fig. 2:**
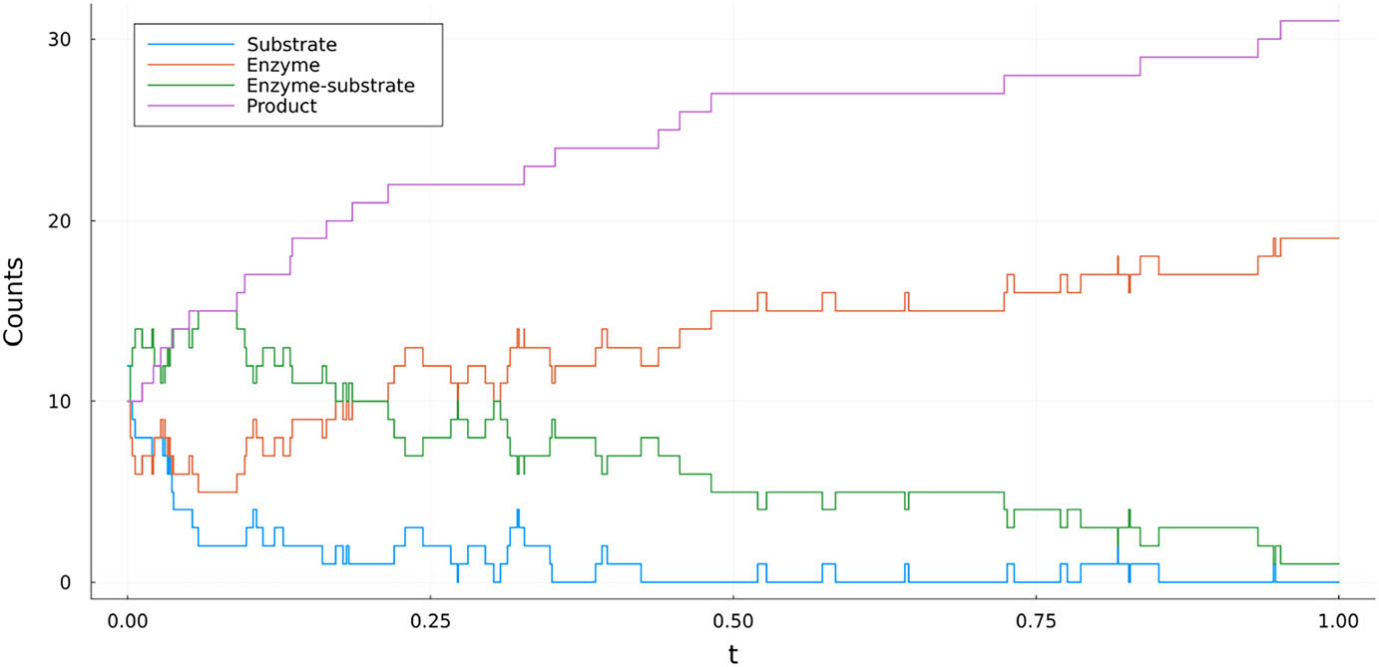
Realization of the forward process of Example 2.4 using $\kappa _1 = 5$, $\kappa _2 = 5$, $\kappa _3=3$ and initial position $x_0 = (12, 10, 10, 10)$

## Guided Markov processes

### First ideas

We first provide some intuition on how the dynamics of a chemical reaction network change upon conditioning on a future event. Let $\mathcal {E}$ denote some event later than time *t*, for example $\{X(T)=v\}$. For $t \in [0,T)$, $\Delta >0$ and $x\in {\mathbb S}$ and $y=x+\xi _\ell$,

$$\begin{aligned} \begin{aligned}&{\mathbb P}\left( X(t+\Delta ) =y\mid X(t)=x, \mathcal {E}\right) = \frac{{\mathbb P}\left( X(t+\Delta )=y,\, \mathcal {E} \mid X(t)=x\right) }{{\mathbb P}\left( \mathcal {E}\mid X(t)=x\right) }\\&\qquad = \frac{{\mathbb P}\left( \mathcal {E}\mid X(t+\Delta )=y,\,X(t)=x\right) {\mathbb P}\left( X(t+\Delta )=y\mid X(t)=x\right) }{{\mathbb P}\left( \mathcal {E}\mid X(t)=x\right) }\\&\qquad = {\mathbb P}\left( X(t+\Delta )=y\mid X(t)=x\right) \frac{{\mathbb P}\left( \mathcal {E}\mid X(t+\Delta )=y\right) }{{\mathbb P}\left( \mathcal {E}\mid X(t)=x\right) } \end{aligned} \end{aligned}$$

Taking the limit $\Delta \downarrow 0$, this suggests that if *X* is conditioned on the event $\mathcal {E}$ then it is a chemical reaction process with adjusted intensities

3.1 $$\begin{aligned} \lambda _\ell ^h(t,x) = \lambda _\ell (t,x)\frac{h(t,x+\xi _\ell )}{h(t,x)}, \end{aligned}$$

where $h(t,x) = {\mathbb P}\left( \mathcal {E}\mid X(t)=x\right)$. The process with intensities $\lambda _\ell ^h(t,x)$ has generator

3.2 $$\begin{aligned} \mathcal {L}^h_t f(x) = \sum _{\ell \in \mathcal {R}} \lambda _\ell ^h(t,x)\left( f(x+\xi _\ell )-f(x)\right) . \end{aligned}$$

To sample the conditioned process, the function *h* is required which is rarely available in closed form. The general idea behind the construction of what we call a guided chemical reaction process is to replace *h* by a suitable tractable substitute *g*. This gives a process with rates $\lambda _t^g$. Discrepancies between the true conditioned process and the guided process can be accounted for by evaluating the likelihood ratio of their measures on path space. In this section we will establish sufficient conditions on *g* for this approach to be valid.

As shown below, conditioning the process corresponds to a change of measure. To see this connection, a direct computation shows that $\mathcal {L}^h_t f$ as defined in (3.2) can be expressed in terms of $\mathcal {L}_t$:

3.3 $$\begin{aligned} \mathcal {L}_t^h f(x) = \frac{1}{h(t,x)} \left[ \mathcal {L}_t (fh)(t,x) - f(x)\mathcal {L}_th(t,x)\right] . \end{aligned}$$

This operator is strongly connected to exponential changes of measure studied in Palmowski and Rolski (2002) and Corstanje et al. (2023) for a wider class of Markov processes.

### Guiding by a change of measure

We assume $x_0$ to be known and that $\mathcal {E}=\{LX(T)=v\}$ is observed for some known $v\in {\mathbb R}^m$ and a matrix $L\in {\mathbb R}^{m\times d}$ of full row rank with $m\le d$. In case $m=d$, we assume without loss of generality that $L=I$. Additionally, we assume $\mathbb {P}(LX(T)=v \mid X(0)=x_0)>0$. The extension to multiple observations will follow in a straightforward way in later sections. Let $\mathcal {A}$ denote the infinitesimal generator of the space-time process (*t*, *X*(*t*)). That is $(\mathcal {A}g)(s,x)=\lim _{\Delta \downarrow 0} \Delta ^{-1} \mathbb {E}\left[ g(s+\Delta , X(s+\Delta )) - g(s,X(s)) \mid X(s) = x\right]$, for those *g* (that map to $\mathbb {R}$) for which the limit exists. Such *g* are said to be in the domain of the generator, denoted by $\mathcal {D}(\mathcal {A})$. While $\mathcal {D}(\mathcal {A})$ is implicitly defined, as part of the definition of $\mathcal {A}$, we can find a more explicit expression on a smaller set of functions. For those functions $\mathcal {A} = \partial _t + \mathcal {L}$, where $\mathcal {L}$ is defined in (2.3). From this, one can see that if there are finitely many reactions, bounded functions that are differentiable with respect to their time-argument are in the domain. For suitable $g:[0,T) \times {\mathbb S} \rightarrow {\mathbb R}$ define

3.4 $$\begin{aligned} E^g(t) = \frac{g(t, X(t))}{g(0,x_0)}\exp \left( -\int _{0}^t \frac{\mathcal {A}g}{g}(s, X(s))\,\textrm{d}s \right) , \qquad t\in [0,T). \end{aligned}$$

#### Definition 3.1

We write $g\in \mathcal {G}$ if $g\in \mathcal {D}(\mathcal {A})$ is a strictly positive function and there exists a filtration $(\mathcal {F}_t,\, t\ge 0)$ such that $(E^g(t),\, t\in [0,T))$ is a martingale adapted to $(\mathcal {F}_t,\, t\ge 0)$.

As $\mathcal {F}_t^X = \sigma (X^t)$ is a sub-$\sigma$-algebra of $\mathcal {F}_t$ for all $t\ge 0$, $(E^g(t),\, t\in [0,T))$ is a martingale with respect to $(\mathcal {F}_t^X,\, t\ge 0)$ as well whenever $g\in \mathcal {G}$. In Lemma 3.3, we give an explicit condition under which $g\in \mathcal {G}$.

Let $g\in \mathcal {G}$ and let ${\mathbb P}_t$ denote the law of the process *X* restricted to $\mathcal {F}_t^X$ for $t\in [0,T)$. Since $(E^g(t),\, t\in [0,T))$ is a martingale with respect to $(\mathcal {F}_t^X,\, t\ge 0)$, we can define a new family of consistent probability measures measures $\{{\mathbb P}_t^g :t\in [0,T)\}$ by

3.5 $$\begin{aligned} \,\textrm{d}{\mathbb P}_t^g = E^g(t) \,\textrm{d}{\mathbb P}_t, \qquad t\in [0,T). \end{aligned}$$

Since $\{{\mathbb P}_t^g :t\in [0,T)\}$ is consistent, there exists a measure ${\mathbb P}_T^g$ such that for all *t*, we have ${\mathbb P}_T^g \big |_{\mathcal {F}_t^X} = {\mathbb P}^g_t$ and, by Theorem 4.2 of Palmowski and Rolski (2002), the process $t\mapsto f(t,X(t)) - \int _0^t \left( \partial _t f+ \mathcal {L}^g_t f \right) (s,X(s)) \,\textrm{d}s$ is a martingale under ${\mathbb P}^g_T$ for any function $f:[0,T)\times {\mathbb S}\rightarrow {\mathbb R}$ finitely supported in *x* and differentiable in *t*. Hence, under $\mathbb {P}^g_T$, *X* is a chemical reaction process with the same jumps as under ${\mathbb P}$ but with adjusted intensities $\lambda ^g_\ell (t,x) = \lambda (t,x) g(t,x+\xi _\ell )/g(t,x)$. For the reader’s convenience we have summarised some of the main arguments of Palmowski and Rolski (2002) for establishing this connection in Appendix D.

#### Definition 3.2

The *guided* process induced by *g* on [0, *t*] is defined as the process *X* under $\mathbb {P}^g_T$.

Some authors refer to this process as the *twisted* process (see e.g. Moral (2017)). Upon denoting the transition probabilities of *X* by *p*, i.e. ${\mathbb P}(X(T) \in A\mid X(t) =x) =\sum _{y\in A} p(t,x; T,y)$ for $A\subseteq {\mathbb S}$, it follows from Example 2.4 of Corstanje et al. (2023) that the conditioned process $\left( X\mid LX(T)=v\right)$ is obtained by taking $g=h$ with

3.6 $$\begin{aligned} h(t,x) = \sum _{\zeta \in L^{-1} v} p(t,x; T, \zeta ), \end{aligned}$$

where $L^{-1}v = \{ y\in {\mathbb S}: Ly=v \}$. We recall our assumption $h(0,x_0)>0$, as otherwise conditioned paths have probability zero. Note that *h* is bounded and satisfies Kolmogorov’s backward equation: $\mathcal {A} h=0$. By Proposition 3.2 in Palmowski and Rolski (2002), $h \in \mathcal {G}$ and thus we can define the probability measure ${\mathbb P}^h_t$ by

$$\begin{aligned} \,\textrm{d}{\mathbb P}_t^h = \frac{h(t,X(t))}{h(0,x_0)} \,\textrm{d}{\mathbb P}_t,\qquad {t \le T}.\end{aligned}$$

Intuitively, ${\mathbb P}_t^h$ gives more mass to paths where *h*(*t*, *X*(*t*)) is large.

Unfortunately, *h* is intractable as the transition probabilities *p* are only known in closed-form in very specific cases including Example 2.2. To resolve this, consider another function $g\in \mathcal {G}$ that acts as a tractable substitute for *h*. Then, as a consequence, the process *X* is tractable and can be simulated under the measure ${\mathbb P}_T^{g}$. Moreover, for $t<T$,

3.7 $$\begin{aligned} {\frac{\,\textrm{d}{\mathbb P}^h_t}{\,\textrm{d}{\mathbb P}^{g}_t}}(X) = \frac{E_t^h}{E_t^{g}} = \frac{h(t,X(t))}{g(t,X(t))}\frac{g(0,x_0)}{h(0,x_0)}\Psi _t^{g}(X), \end{aligned}$$

where

3.8 $$\begin{aligned} \Psi _t^{g}(X) = \exp \left( \int _0^t \frac{\mathcal {A}g}{g}(s,X(s))\,\textrm{d}s\right) . \end{aligned}$$

To evaluate $\Psi ^g$, a direct computation yields

3.9 $$\begin{aligned} \frac{\mathcal {A}g}{g}(s,x) = \partial _s \log g(s,x) + \sum _{\ell \in \mathcal {R}} \left( \lambda _\ell ^{g}(s,x) - \lambda _\ell (s,x)\right) . \end{aligned}$$

Eq. 3.7 becomes particularly useful if it can be evaluated in $t=T$ as well. As $h(T,x) = \textbf{1}_{\{Lx=v\}}$, this only depends on the choice for *g*. We present a class of functions that can subsequently be used for this purpose in Theorem 3.4.

#### Lemma 3.3

Suppose $g\in \mathcal {D}(\mathcal {A})$ is a strictly positive function such that for some positive constant *C*

$$\begin{aligned} \int _0^T \sum _{\ell \in \mathcal {R}} \lambda _\ell (s,X(s))\left( \frac{g(s,X(s)+\xi _\ell )}{g(s,X(s))}-1\right) ^2 \,\textrm{d}s < C,\end{aligned}$$

$\mathbb {P}$-almost surely, then $g\in \mathcal {G}$.

#### Proof

The proof is inspired by the proof given in Example 15.2.10 in Brémaud (2020) and Lemma 19.6 in Liptser and Shiryaev (2013).

It suffices to show that ${\mathbb E}\left[ E^g(T)\right] =1$. By Lemma 3.1 of Palmowski and Rolski (2002), $E^g$ is a local martingale. Let $\left\{ \sigma _n\right\} _n$ be a localizing sequence for $E^g$. Then, if $(E^g(t),\, t\in [0,T])$ were uniformly integrable, then

$$\begin{aligned} {\mathbb E}\left[ E^g(T)\right] = \lim _{n\rightarrow \infty }{\mathbb E}\left[ E^g(T\wedge \sigma _n)\right] = {\mathbb E}\left[ E^g(0)\right] =1\end{aligned}$$

We proceed to show uniform integrability. Given $t\in [0,T]$, suppose a trajectory *X* has jumps at times $t_1,\dots ,t_N$. Set $t_0=0$ and $t_{N+1}=t$. Then

3.10 $$\begin{aligned} \begin{aligned} E^g(t)&= \frac{g(t,X(t))}{g(0,x_0)}\exp \left( -\sum _{j=0}^N\int _{t_j}^{t_{j+1}}\partial _s \log g(s, X(t_j))\,\textrm{d}s - \int _0^t \frac{\mathcal {L}g(s,X(s))}{g(s,X(s))}\,\textrm{d}s \right) \\&= \frac{g(t,X(t))}{g(0,x_0)}\prod _{j=0}^N\frac{g(t_j,X(t_j))}{g(t_{j+1},X(t_j))} \exp \left( -\int _0^t\frac{\mathcal {L}g(s,X(s))}{g(s,X(s))}\,\textrm{d}s \right) \\&= \prod _{j=0}^{N-1}\frac{g(t_{j+1},X(t_{j+1}))}{g(t_{j+1},X(t_j))} \exp \left( -\int _0^t \frac{\mathcal {L}g(s,X(s))}{g(s,X(s))}\,\textrm{d}s\right) \\&= \prod _{j=0}^{N-1}\frac{g(t_{j+1},X(t_{j+1}))}{g(t_{j+1},X(t_j))} \exp \left( -\int _0^t \sum _{\ell \in \mathcal {R}} \lambda _\ell (s,X(s))\left( \frac{g(s,X(s)+\xi _\ell )}{g(s,X(s))}-1\right) \,\textrm{d}s\right) \end{aligned} \end{aligned}$$

From (3.10), it is easy to verify that

$$\begin{aligned} E^g(t)^2 = E^{g^2}(t)\exp \left( \int _0^t \sum _{\ell \in \mathcal {R}} \lambda _\ell (s,X(s))\left( \frac{g(s,X(s)+\xi _\ell )}{g(s,X(s))} -1 \right) ^2 \,\textrm{d}s \right) \end{aligned}$$

By Lemma 3.1 of Palmowski and Rolski (2002), $E^{g^2}$ is a local martingale and, since it is bounded from below, it is a supermartingale with ${\mathbb E}\left[ E^{g^2}(t)\right] \le 1$. Hence, for all *n*,

$$\begin{aligned}\begin{aligned}&\mathbb {E}\left[ E^g(T\wedge \sigma _n)^2\right] \\&\le {\mathbb E}\left[ E^{g^2}(T\wedge \sigma _n)\exp \left( \int _0^T \sum _{\ell \in \mathcal {R}} \lambda _\ell (s,X(s))\left( \frac{g(s,X(s)+\xi _\ell )}{g(s,X(s))} -1 \right) ^2 \,\textrm{d}s \right) \right] \le e^C \end{aligned} \end{aligned}$$

Therefore $$\sup _n \mathbb {E}\left[ E^g(T\wedge \sigma _n)^2\right] <\infty $$. $$\square $$

#### [Style2 Style2]Theorem 3.4

Define *h* is as in (3.6) and let $g:[0,T]\times {\mathbb S}\rightarrow {\mathbb R}$ be such that the condition of Lemma 3.3 is satisfied. Then

3.11 $$\begin{aligned} \frac{\,\textrm{d}{\mathbb P}^h_T}{\,\textrm{d}{\mathbb P}^g_T} = \frac{g(0,x_0)}{h(0,x_0)}\frac{\Psi _T^g(X)}{g(T,X(T)}\textbf{1}_{\{LX(T)=v\}}. \end{aligned}$$

#### Proof

The form of the Radon-Nikodym derivative follows from (3.7) upon noting that for $t\uparrow T$,

$$\begin{aligned} h(t,X(t)) = {\mathbb E}\left[ \textbf{1}\{ LX(T)=v\} \mid \mathcal {F}_t\right] \rightarrow {\mathbb E}\left[ \textbf{1}\{ LX(T)=v\} \mid \mathcal {F}_T\right] = \textbf{1}\{LX(T)=v\}. \end{aligned}$$

$$\square $$

The following proposition is often helpful in establishing that the condition of Lemma 3.3 is satisfied for a given *g*.

#### Proposition 3.5

Suppose $g:[0,T]\times {\mathbb S}\rightarrow {\mathbb R}$ is such that for all *x* the map $t\mapsto g(t,x)$ is bounded from above and bounded away from zero. Then the condition of Lemma 3.3 is satisfied.

#### Proof

Since we assume throughout *X* to be nonexplosive, we have that almost surely the maps $t\mapsto g(t,X(t)+\xi _\ell )/g(t,X(t))$ are almost surely bounded on [0, *T*] for all $\ell \in \mathcal {R}$. The result now follows upon noting that $\int _0^T \sum _{\ell \in \mathcal {R}} \lambda _\ell (s,X(s))\,\textrm{d}s <\infty$ by Assumption (2.1c). $\square$

#### Corollary 3.6

The likelihood of $x_0$ based on the observation *v* is given by

3.12 $$\begin{aligned} h(0,x_0) = g(0,x_0) {\mathbb E}_T^{g}\left[ \frac{\Psi _T^{{g}}(X)}{g(T,X(T))}\textbf{1}_{\{LX(T)=v\}}\right] . \end{aligned}$$

#### Proof

This follows immediately upon integrating (3.11) with respect to ${\mathbb P}_T^{g}$. $\square$

Note that $g\equiv 1$ satisfies the condition in Lemma 3.3. This choice simply yields the original forward process. Sampling the conditioned process in this way is however very inefficient when ${\mathbb P}\left( LX(T)=v\right)$ is low.

The lemma below shows that the law of the guided process does not change when *g* is multiplied by a function only depending on time.

#### Lemma 3.7

(Invariance under time scaling) Suppose $g\in \mathcal {G}$ and let $c:[0,T]\rightarrow {\mathbb R}_+$ be a differentiable function. Then $E^{cg}(t) = E^{g}(t)$.

#### Proof

Since *c* only depends on time

$$\begin{aligned} \begin{aligned} \int _0^t \frac{\mathcal {A}\left( cg\right) }{cg}(s,X(s))\,\textrm{d}s&= \int _0^t \left[ \partial _s\log \left( c(s)g(s,X(s))\right) + \frac{\mathcal {L}_s\left( cg\right) }{cg}(s,X(s)) \right] \,\textrm{d}s\\&= \int _0^t \partial _s \log c(s)\,\textrm{d}s + \int _0^t \left[ \partial _s\log g(s,X(s)) + \frac{\mathcal {L}_sg}{g}(s,X(s))\right] \,\textrm{d}s\\&= \log c(t)- \log c(0) + \int _0^t \frac{\mathcal {A}g}{g}(s,X(s)) \,\textrm{d}s. \end{aligned}. \end{aligned}$$

From this, we get $E^{cg}(t)$ by negating the right-hand-side, taking the exponent and subsequently multiplying by $c(t) g(t,X(t)) /(c(0)g(0,x_0))$. It is easily seen that the terms with *c* cancel out. $\square$

The following proposition is useful for numerical evaluation of the likelihood of a sampled guided process.

#### Proposition 3.8

Suppose $$0=t_0< t_1< \cdots<t_{N-1}<t_N=T$$, $$x_0,\dots , x_N\in {\mathbb S}$$ and

$$\begin{aligned} x(t) = \sum _{j=0}^{N-1} x_j \textbf{1}_{[t_j,t_{j+1})}(t). \end{aligned}$$

Then, with $$\alpha _j(s):=g(s,x_{j+1})/g(s,x_j)$$

$$\begin{aligned} \begin{aligned}&\frac{g(0,x_0)}{g(T, x(T))} \exp \left( \int _0^T \frac{\mathcal {A}g}{g}(s,x(s))\,\textrm{d}s \right) \\ &\qquad = \left( \prod _{j=0}^{N-2} \frac{1}{\alpha _j(t_{j+1})} \right) \exp \left( \sum _{j=0}^{N-1} \int _{t_j}^{t_{j+1}} \sum _{\ell \in \mathcal {R}} \lambda _\ell (s, x_j)\left[ \alpha _j(s) - 1\right] \,\textrm{d}s \right) \end{aligned} \end{aligned}$$

#### Proof

This is a consequence of (3.10). $\square$

## Choices for *g*

It remains to specify the maps $(t,x) \mapsto g(t,x)$ satisfying the assumption of Lemma 3.3. Moreover, to be of any use, the event $\{LX(T)=v\}$ needs to get positive probability under ${\mathbb P}^g_T$.

In this section we show the following:

- Sect. 4.1: The choices for *g* in Fearnhead (2008) and Golightly and Sherlock (2019), both based on the chemical Langevin equation, lead to absolute continuity.
- Sect. 4.2: A guiding function based on the transition density of a scaled Brownian motion yields absolute continuity while being computationally very efficient. It satisfies ${\mathbb P}^g_T(LX(T)=v)>0$. In subsection 4.2.1 we show how *g* can be extended to the case of multiple partial observations.
- Sect. 4.3: For processes with monotone components a specific choice for *g* can overcome certain problems arising in the aforementioned choices.

In the following, *C* denotes a positive definite matrix.

### Choices based on the chemical Langevin equation

Consider the CLE given in (2.5). Set $a_{\textrm{CLE}}={\sigma _{\textrm{CLE}}}{\sigma _{\textrm{CLE}}}^{\prime }$. Fearnhead (2008) proposed to choose *g* based on the Euler discretization of the CLE. This gives

4.1 $$\begin{aligned} g_\textrm{F}(t,x) = \mathcal {N}\left( v; L(x+b_{\textrm{CLE}}(t,x)(T-t)), La_{\textrm{CLE}}(t,x)L^{\prime }(T-t) + C\right) , \end{aligned}$$

As the assumption of Proposition 3.5 is satisfied we obtain ${\mathbb P}^h_T \ll {\mathbb P}^{g_{\textrm{F}}}_T$.

Golightly and Sherlock (2019) propose to infer *g* by using the Linear Noise Approximation (LNA) to the CLE. Here, we discuss their specific choice called “LNA with restart”. For notational convenience, write $b=b_{\textrm{CLE}}$ and $\sigma =\sigma _{\textrm{CLE}}$ and again set $a=\sigma \sigma ^{\prime }$. Given $t<T$ and $x\in {\mathbb S}$, we denote by $z_{T\mid (t,x)}$ and $V_{T\mid (t,x)}$ the solutions at time *T* of the system of ordinary differential equations

$$\begin{aligned} \begin{aligned} \,\textrm{d}z_{s\mid (t,x)}&= {b}(s, z_{s\mid (t,x)}) \,\textrm{d}s \\ \,\textrm{d}V_{s\mid (t,x)}&=\left( V_{s\mid (t,x)}\left( J_{b}(s, z_{s\mid (t,x)})\right) ^{\prime }+ J_{b}\left( s,z_{s\mid (t,x)}\right) V_{s\mid (t,x)}+{a}\left( s, z_{s\mid (t,x)}\right) \right) \,\textrm{d}s \end{aligned} \end{aligned}$$

where $s \in [t,T]$, subject to the initial conditions are given by $z_{t\mid (t,x)} = x$ and $V_{t\mid (t,x)} = 0$. Here, $J_{b}$ denotes the Jacobian matrix of *b* (having component (*i*, *j*) given by $\partial b_i / \partial z_j$. The guiding term then is given by

4.2 $$\begin{aligned} g_{\textrm{LNAR}}(t,x) = \mathcal {N}\left( v; L z_{T\mid (t,x)}, LV_{T\mid (t,x)}L^{\prime }+C\right) . \end{aligned}$$

Absolute continuity $\mathbb {P}^h_T \ll \mathbb {P}^{g_{\textrm{LNAR}}}_T$ follows by the same argument used for $g_{\textrm{F}}$.

### Choosing *g* using the transition density of a scaled Brownian motion

For fixed $\sigma \in {\mathbb R}^{d\times d}$ define the process $\tilde{X}$ by $\,\textrm{d}\tilde{X}(t) = {\sigma }\,\textrm{d}W(t)$, where *W* is a standard Brownian motion. Denote ${a}={\sigma }{\sigma }^{\prime }$ and assume that *a* is such that $L{a}L^{\prime }$ is strictly positive definite. We derive *g* from backward filtering the process $\tilde{X}$ with observation $V\mid \tilde{X}(T) \sim \mathcal {N}(L\tilde{X}(T),C)$. Let *q* denote the density of the $\mathcal {N}(0,C)$-distribution. The density of *V*, conditional on $\tilde{X}(t)=x$ is given by $u(t,x):=\int \tilde{p}(t,x;T,y)q(v-Ly)\,\textrm{d}y$. It follows from the results in Mider et al. (2021) that $u(t,x)\propto g(t,x)$ where

4.3 $$\begin{aligned} g(t,x)= \exp \left( -\frac{1}{2} x^{\prime }H(t) x + F(t)^{\prime }x\right) . \end{aligned}$$

Here, for $t\le T$, *H*(*t*) and *F*(*t*) satisfy the ordinary differential equations

4.4 $$\begin{aligned} \begin{aligned} \,\textrm{d}H(t)&= H(t){a} H(t) \,\textrm{d}t, \qquad H(T) = L^{\prime }C^{-1} L \\ \,\textrm{d}F(t)&= H(t) {a} F(t)\,\textrm{d}t,\qquad F(T)=L^{\prime }C^{-1}v\end{aligned}. \end{aligned}$$

These differential equations can be solved in closed-form:

4.5 $$\begin{aligned} H(t) = z(t)H(T) \quad \text {and} \quad F(t) = z(t) F(T), \end{aligned}$$

with $$z(t)=\left( I+H(T){a}(T-t)\right) ^{-1}$$

Note that we have not yet specified $\sigma$. Depending on its choice, *g* may be radically different from *h*. Nevertheless, it can be used as a change of measure to condition paths of *X* on the event $\{LX(T)=v\}$.

#### [Style2 Style2]Theorem 4.1

Let *g* be defined by (4.3) and (4.4). Then ${\mathbb P}_T^h\ll {\mathbb P}_T^{g}$ with

4.6 $$\begin{aligned} {\frac{\,\textrm{d}{\mathbb P}_T^h}{\,\textrm{d}{\mathbb P}_T^{g}}} = \frac{g(0,x_0)}{h(0,x_0)} \exp \left( \int _0^T \frac{\mathcal {A}g}{g} (s,X(s))\,\textrm{d}s \right) \exp \left( -\frac{1}{2} v^{\prime }C^{-1}v\right) \textbf{1}_{\{LX(T)=v\}}. \end{aligned}$$

#### Proof

It follows from theorems 2.4 and 2.5 in Mider et al. (2021) that if we define

$$\begin{aligned} M(t):= \left( C+L{a}L^{\prime }(T-t)\right) ^{-1}, \end{aligned}$$

then $H(t) = L^{\prime }M(t)L$ and $F(t) =L^{\prime }M(t)v$ and therefore

4.7 $$\begin{aligned} g(t,x) \propto \exp \left( -\frac{1}{2} (v-Lx)^{\prime }M(t)(v-Lx) \right) . \end{aligned}$$

The proportionality is up to a differentiable time-dependent function and hence does not affect the change of measure by Lemma 3.7. By Lemma C.1, *g* is bounded from above and bounded away from 0. Since *g* is smooth in *t* and well-defined in *T*, we clearly have continuous differentiability of the maps $t\mapsto g(t,x)$ for all $x\in {\mathbb S}$ on [0, *T*]. The result thus follows from Proposition 3.5 and Theorem 3.4. $\square$

Theorem 4.1 is only of interest when the guided process has a positive probability of hitting *v* at time *T*. This is ensured by Theorem 4.2 for which the proof is deferred to Appendix A.

#### [Style2 Style2]Theorem 4.2

$${\mathbb P}^{g}(LX(T)=v)>0$$

A convenient choice for the matrix *C* has computational advantages.

#### Definition 4.3

Let $\varepsilon >0$ and set $C = \varepsilon L{a}L^{\prime }$. The corresponding *g* in (4.3) is denoted by $g_\varepsilon$.

#### Lemma 4.4

The induced measure $\mathbb {P}^{g_\varepsilon }_T$ is the law of a chemical reaction process with intensities $\lambda _\ell ^{g_\varepsilon }(t,x)=\alpha ^{g_\varepsilon }_\ell (t,x) \lambda _\ell (t,x)$, where

4.8 $$\begin{aligned} \alpha ^{g_\varepsilon }_\ell (t,x) = \exp \left( - \frac{ d(v, L(x+\xi _\ell ))^2-d(v,Lx)^2}{2(\varepsilon + T-t )} \right) , \end{aligned}$$

with *d* the metric given by

4.9 $$\begin{aligned} d(x,y)^2 = (y-x)^{\prime }(LaL^{\prime })^{-1}(y-x). \end{aligned}$$

#### Proof

This result follows by substituting the expression for *g* in (4.7) into the definition of $\alpha ^g_\ell (t,x)$ and rewriting in terms of the metric *d*. $\square$

This implies that only one matrix inverse, $(LaL^{\prime })^{-1}$, needs to be calculated. By Eq. 4.8, the intensity of the guided process either becomes very large or small depending on whether $L(x+\xi _\ell )$ is closer or further away from *v* than *Lx* with respect to the metric *d*, which has intuitive appeal. Also note that $\varepsilon$ appears only in the denominator at $T+\varepsilon$, which implies that the choice $C=\varepsilon L{a}L^{\prime }$ imposes the conditioning at at time $T+\varepsilon$ instead of *T*, thereby precluding explosive behaviour in (4.7) as $t\uparrow T$.

#### Remark 4.5

Ideally, we would like to have ${\mathbb P}^{g}\left( LX(T)=v\right) =1$. For $g=g_\varepsilon$ as in Lemma 4.4 this is not guaranteed. Essentially, the choice $C=\varepsilon L{a}L^{\prime }$ yields a process where *LX*(*t*) hits *v* at time $T+\varepsilon$. Since the intensities are bounded from below and above, there is a positive probability of at least one reaction occurring in $(T,T+\varepsilon )$ resulting in $LX(T)\ne v$. In Sect. 5 we discuss the case where $\varepsilon =0$, which does yield ${\mathbb P}^{g}(LX(T)=v)=1$.

#### Remark 4.6

Comparing $g_{\textrm{F}}$, $g_{\textrm{LNAR}}$ and $g_\varepsilon$, it is likely that $g_\varepsilon$ will deviate most from *h*. In particular when the drift and diffusion coefficient of the CLE are nonlinear, $g_\varepsilon$ will likely deviate from *h* the most leading to the guided paths deviating from the conditioned paths. However, from a computational point of view $g_\varepsilon$ is most attractive, as can be seen from (4.8). Moreover, in Sect. 6 it turns out that simulation of the guided process is simplified in case of $g_\varepsilon$.

#### Remark 4.7

In dimension 1, we derive a better intuition for the choice for *a* from (4.8) and (4.9). A small choice leads to a higher guided intensity, and thus a process arriving around *v* quickly and likely to stay near *v*, while a large value of *a* leads to trajectories that remain unaffected by $\alpha _\ell ^{g_\varepsilon }$ until *t* approaches *T*. Ideally, we mimic trajectories under ${\mathbb P}^h$ and thus a good choice of *a* ensures $g(0,x_0)\Psi _T^g(X)\textbf{1}\{LX(T)=v\}/g(T,X(T))$ is large (with $\Psi _t^g(X)$ defined in Equation (3.8)). This can be verified using Monte-Carlo simulation sampling *X* under ${\mathbb P}^{g_\varepsilon }$. Alternatively, a simple choice for *a* is $a_{\textrm{CLE}}(0,x_0)$ or, if we have a complete observation, $a=a_{\textrm{CLE}}(T,v)$.

#### Extension to multiple observations

We now extend Theorem 4.1 to a result for multiple observations. Consider observations $v_i = L_i X(t_i)$, $i=1,\dots ,n$ where $0=t_0<t_1<\cdots <t_n$ and assume without loss of generality that $L_i \in {\mathbb R}^{m_i\times d}$ are of full column rank with $m_i\le d$ and $L_i = I$ when $m_i=d$.

##### Proposition 4.8

Let *p* denote the transition probabilities of *X* and define for $t\in [t_{k-1},t_k)$ and $x\in {\mathbb S}$

4.10 $$\begin{aligned} \begin{aligned} h(t,x)&= {\mathbb P}\left( L_iX(t_i)=v_i,\, i=k,\dots ,n\mid X(t)=x\right) \\&= \sum _{\zeta _k \in L_k^{-1}v_k} \cdots \sum _{\zeta _n \in L_n^{-1}v_n} p(t,x;t_k, \zeta _k)\prod _{i=k}^{n-1} p(t_i,\zeta _i;t_{i+1},\zeta _{i+1}). \end{aligned} \end{aligned}$$

Then $h\in \mathcal {G}$ and the change of measure (3.5) induces $\left( X\mid L_kX(t_k)=v_k,\, k=1,\dots ,n\right)$.

##### Proof

This result is obtained upon following Example 2.4 of Corstanje et al. (2023) using the delta-dirac distribution $\mu (\zeta _k) = \delta (v_k-L_k\zeta _k)$. $\square$

We deduce the form of *g* from Mider et al. (2021) in a similar way compared to a single observation. We consider an auxiliary process $\tilde{X}$ that solves the SDE

4.11 $$\begin{aligned} \,\textrm{d}\tilde{X}(t) = {\sigma }(t) \,\textrm{d}W(t), \qquad X(0)=x_0,\, t\in [0,t_n), \end{aligned}$$

where ${\sigma }(t) = \sum _{k=1}^{n}{\sigma }_k\textbf{1}_{[t_{k-1},t_k)}(t)$ and ${a}_k = {\sigma }_k{\sigma }_k^{\prime }$ are positive definite $d\times d$ matrices for $k=1,\dots ,n$.

For each observation *k*, we consider $V_k\mid X(t_k)\sim \mathcal {N}(0,C_k)$ where $C_k$ is an $m_k\times m_k$ covariance matrix. Suppose $q_k$ denotes the density of the $\mathcal {N}(0,C_k)$-distribution. Then the transition density of $\tilde{X}$ satisfies for $t\in [t_{k-1},t_k)$,

4.12 $$\begin{aligned} \begin{gathered} \int \tilde{p}(t,x;t_k,\zeta _k)q_k(v_k-L_k\zeta _k)\prod _{i=k}^{n-1}\tilde{p}(t_i,\zeta _i;t_{i+1},\zeta _{i+1})q_{i+1}(v_{i+1}-L_{i+1}\zeta _{i+1}) \,\textrm{d}\zeta _k\cdots \,\textrm{d}\zeta _n \\ \propto \exp \left( -\frac{1}{2} x^{\prime }H(t)x + F(t)^{\prime }x\right) =: g(t,x). \end{gathered} \end{aligned}$$

Here, the expressions for *H* and *F* can be found by backward solving a system of equations given by

$$\begin{aligned} \begin{aligned} H(t_n)&= L_n^{\prime }C_n^{-1}L_n\\ F(t_n)&= L_n^{\prime }C_n^{-1}v_n \end{aligned} \end{aligned}$$

and for $$t\in (t_{k-1},t_k)$$, $$k=1,\dots ,n$$

4.13 $$\begin{aligned} \begin{aligned} \,\textrm{d}H(t)&= H(t)a_k H(t) \,\textrm{d}t, \qquad H(t_k) = H_k:= L_k^{\prime }C_k^{-1}L_k + H(t_k+) \\ \,\textrm{d}F(t)&= H(t)a_k F(t) \,\textrm{d}t, \qquad F(t_k) = F_k:= L_k^{\prime }C_k^{-1}v_k + F(t_k+) \end{aligned} \end{aligned}$$

Finally, we let *H* and *F* be a right continuous modification of the solution to (4.13), i.e. setting $H(t_k) = H(t_k+)$ and $F(t_k)=F(t_k+)$, $k=1,\dots ,n-1$. This system can be solved in closed form: ${H}(t) = z_k(t)H_k$, ${F}(t) = z_k(t)F_k$, where, for $k=1,\dots ,n$, $z_k(t) = \left( I+H_ka_k(t_k-t)\right) ^{-1}$.

##### Remark 4.9

Alternatively, following the computations in Section 2 of Mider et al. (2021), *g* can be expressed as

4.14 $$\begin{aligned} g(t,x) \propto \exp \left( -\frac{1}{2} \left( v(t)-L(t)x\right) ^{\prime }M(t)\left( v(t)-L(t)x\right) \right) , \end{aligned}$$

where, the proportionality is up to a time-dependent function and for $t\in [t_{k-1},t_k)$

$$\begin{aligned} L(t) = \begin{pmatrix} L_k \\ \vdots \\ L_n \end{pmatrix} \qquad \text {and}\qquad v(t) = \begin{pmatrix} v_k \\ \vdots \\ v_n \end{pmatrix} \end{aligned}$$

and $M(t) = M^\dagger (t)^{-1}$ with $M^\dagger (t)$ a block matrix with entries

$$\begin{aligned} M^\dagger (t) = \begin{pmatrix} C_i\textbf{1}\{i=j\} +\sum _{l=k}^{i\wedge j-1} L_i a_{l+1} L_j^{\prime }(t_{l+1}-t_{l}) + L_i a_{k}L_j^{\prime }(t_{k}-t)\end{pmatrix}_{i,j=k}^n, \end{aligned}$$

$t\in [t_{k-1},t_k)$. While this representation of *g* is useful in most proofs, it is computationally more demanding as the matrix dimensions of *H*(*t*) and *F*(*t*) are $d\times d$, while *M*(*t*), *L*(*t*) and *v*(*t*) have dimensions that increase with the amount of observations.

Using Lemma C.2, the proof of Theorem 4.1 can be repeated for a result like Theorem 4.1 for each observation *k* to find that ${\mathbb P}_{t_n}^h \ll {\mathbb P}_{t_n}^{g}$ with

4.15 $$\begin{aligned} {\frac{\,\textrm{d}{\mathbb P}_{t_n}^h}{\,\textrm{d}{\mathbb P}_{t_n}^{g}}} = \frac{g(0,x_0)}{h(0,x_0)} \exp \left( \int _0^{t_n}\frac{\mathcal {A}g}{g}(s,X(s))\,\textrm{d}s - \frac{1}{2} \sum _{k=1}^n v_k^{\prime }C_k^{-1}v_k\right) \textbf{1}_{A_n}. \end{aligned}$$

The term $\exp \left( - \frac{1}{2} \sum _{k=1}^n v_k^{\prime }C_k^{-1}v_k\right)$ results from evaluating each fraction $g(t_k, x)/g(t_k-,x)$ on the set $\left\{ L_k x=v_k\right\}$. We can repeat Theorem 4.2 for each observation to obtain ${\mathbb P}^{g}\left( A_n \right) >0$. However, similar to Remark 4.5, we generally do not have ${\mathbb P}^{g}\left( A_n \right) = 1$.

### Choosing *g* to guide a process with monotone components

Theorem 4.1 shows that choosing *g* using the transition density of a scaled Brownian motion yields a process that is absolutely continuous with respect to the conditioned process. However, depending on the network, this might not always be a desirable choice.

Consider for instance Example 2.4. Here, the fourth component of the process (*P*) is monotonically increasing as it only appears in reaction 3, where 1 is added, which can also be seen in Fig. 2. Let us for simplicity assume a complete observation at time *T*, that is $X(T)=v_T$. Clearly, if $X_4(t)=v_{T,4}$ for some $t<T$, reaction 3 cannot occur anymore for the process to satisfy the conditioning. However, $\lambda _3^{g}(t,X(t)) \ne 0$ for the choices of *g* discussed so far. This can lead to trajectories that don’t satisfy the conditioning, which in turn means a low acceptance ratio when sampling.

Alternatively, we choose an auxiliary process $\tilde{X} = ( \tilde{Z}, \tilde{Y})$, where $\tilde{Z}$ is the ${\mathbb R}^3$-valued process that solves $\,\textrm{d}\tilde{Z}(t) = \sigma \,\textrm{d}W_t$ and $\tilde{Y}$ is a homogeneous Poisson process with intensity $\tilde{\theta }$. We stick with the notation $x = (z,y)\in {\mathbb R}^4$ with $z\in {\mathbb R}^3$ and $y\in {\mathbb R}$, and denote $v_T = (z_T,y_T)$. Similar to earlier computations, we deduce that

4.16 $$\begin{aligned} g(t,(z,y)) = \exp \left( -\frac{d(z_T,z)^2}{2(T +\epsilon -t)} \right) \frac{\left( \tilde{\theta }(T-t)\right) ^{y_T-y}}{(y_T-y)!}\exp \left( -\tilde{\theta }(T-t)\right) , \end{aligned}$$

where $$\epsilon >0$$ and

$$\begin{aligned} d(z_T,z) = \sqrt{(z_T-z)^{\prime }a^{-1}(z_T-z)}. \end{aligned}$$

This choice satisfies the assumption of Lemma 3.3.

## Choosing *g* so that ${\mathbb P}^h$ and ${\mathbb P}^{g}$ are equivalent

By (4.15), the measures ${\mathbb P}^h$ and ${\mathbb P}^{g}$ are equivalent when ${\mathbb P}^{g}\left( A_n\right) = 1$, as all other terms in the Radon-Nikodym derivative are nonzero. In this section we propose a choice for *g* which yields equivalence of the measures ${\mathbb P}^h$ and ${\mathbb P}^{g}$ under an additional assumption on the network.

By Remark 4.5, the choice $C = \varepsilon LaL^{\prime }$ can be interpreted as imposing a condition of hiting *v* at time $T+\varepsilon$. Therefore, intuitively, equivalence can be achieved upon artificially setting all elements in the matrices $C_k$ equal to 0. In this case, $g(t_k,x)$ becomes ill-defined whenever $L_kx\ne v_k$ and boundedness of *g* is lost, rendering the earlier proofs invalid. To utilize this choice for *g* and show equivalence, we build on earlier work in Corstanje et al. (2023). Proofs of results in this section are given in Appendix B.

### Absolute continuity

The initial conditions for the differential equations (4.13) for the functions *H* and *F* appearing in (4.12) are not defined as the matrices $C_1,\dots ,C_n$ are not invertible, but we can still obtain an equivalent of (4.14). Observe that for $t\in [t_{k-1},t_k)$,

5.1 $$\begin{aligned} \begin{gathered} \int \tilde{p}(t,x;t_k,\zeta _k)\delta _{v_k}(L_k\zeta _k) \prod _{i=k}^{n-1} \tilde{p}(t_i,\zeta _i;t_{i+1},\zeta _{i+1})\delta _{v_{i+1}}(L_{i+1}\zeta _{i+1}) \,\textrm{d}\zeta _k \cdots \,\textrm{d}\zeta _n \\ \propto \exp \left( - \frac{1}{2} (v(t)-L(t)x)^{\prime }M(t)(v(t)-L(t)x)\right) =: g(t,x), \end{gathered} \end{aligned}$$

where *L*, *v* and *M* are defined in Remark 4.9 but with $C_1,\dots ,C_n=0$ and we define $g(t_k,x) = g(t_k+,x)$ for $x\in L_k^{-1}v_k$.

#### [Style2 Style2]Theorem 5.1

Let *g* be defined by (5.1). Then ${\mathbb P}^h \ll {\mathbb P}^{g}$ with

5.2 $$\begin{aligned} {\frac{\,\textrm{d}{\mathbb P}_{t_n}^h}{\,\textrm{d}{\mathbb P}_{t_n}^{g}}} = \frac{g(0,x_0)}{h(0,x_0)} \exp \left( \int _0^{t_n} \frac{\mathcal {A}g}{g}(s,X(s))\,\textrm{d}s\right) \textbf{1}_{A[X^{t_n}]}, \end{aligned}$$

where

$$\begin{aligned} A[X^{t_n}] = \left\{ \sup _{0\le s<t_n} \sum _{\ell \in \mathcal {R}} \lambda _{\ell }^{g}(s,X(s)) <\infty \right\} . \end{aligned}$$

To show that ${\mathbb P}^{g}_{t_n}(A[X^{t_n}])>0$, we require Proposition 5.2, combined with the observation that the proof of Theorem 4.2 can be repeated with this choice for *g* upon observing that $\sum _{\ell \in \mathcal {R}}\lambda _\ell ^{g}$ stays finite on the sets $\{L_kx=v_k\}$.

#### Proposition 5.2

$A_n \subseteq A[X^{t_n}]$ with $A_n$ as defined in (1.3).

#### Remark 5.3

To see that the reverse inclusion generally does not hold, consider a process conditioned to hit *v* at time *T*. If $LX(t)=u\ne v$ for $t\in (T-\varepsilon ,T]$ where *u* is such that no reactions exist such that $d(v,L(u+\xi _\ell )) < d(v,Lu)$, it can be shown that $t\mapsto \frac{g(t,u+\xi _\ell )}{g(t,u)}$ is bounded and therefore such trajectories are included in $A[X^T]$.

### Equivalence

Lemma 5.1 shows that ${\mathbb P}^h$ is absolutely continuous with respect to ${\mathbb P}^{g}$ on the set $A[X^{t_n}]$. For simulation purposes, equivalence would be preferable. It follows from (5.2) that this is indeed the case if ${\mathbb P}^{g}\left( A[X^{t_n}]\right) =1$. This can be shown if the network also satisfies a greedy property.

#### Assumption 5.4

For $k=1,\dots ,n$, define the metric $d_k$ on ${\mathbb R}^{m_k}$ through (B.6) and suppose that $d_1,\dots , d_n$ are such that for all *k*, $t\in [t_{k-1},t_k)$ $x\in {\mathbb S}\setminus L_k^{-1}v_k$, there is a reaction $\ell \in \mathcal {R}$ such that $\lambda _\ell (t,x)>0$ and $d_k(v_k,L_k(x+\xi _\ell ))<d_k(v_k, L_k x)$.

Intuitively, Assumption 5.4 is satisfied when there is always a reaction available that takes the process closer to the first desired conditioning given being in state *x* at time *t*. The choice for *g* will then guarantee that eventually, the path will jump to this point closer to the conditioned state and will therefore hit the observation in finitely many reactions. This argument is formalized in Theorem 5.5.

#### [Style2 Style2]Theorem 5.5

Suppose Assumption 5.4 is satisfied. Then ${\mathbb P}^g_{t_n}(A_n)=1$.

#### Corollary 5.6

It follows from Proposition 5.2 and Theorem 5.5 that, under Assumption 5.4, ${\mathbb P}^{g}_{t_n}(A[X^{t_n}])=1$.

The choice for *g* described in this section has two advantages. The first being that the set $A[X^{t_n}]$ on which absolute continuity is obtained is larger than $A_n$ and the second being that an additional assumption yields equivalence. However, a representation such as (4.12) is not available in this case. When evaluating (4.12), the matrix products and inversions are computed for matrices of size $d\times d$, while (4.14) requires matrix computations where the size increases with the amount of observations. We thus conclude that from a theoretical point of view we may prefer the construction in this section, but for computational purposes (4.12) should be preferred, especially in case *n* is large.

## Simulation methods

In this section we discuss methods for simulating the guided process on [0, *T*]. This is a Markov jump process with time-dependent intensity. In case $\Vert LX(t)-v\Vert$ is large for *t* close to *T*, this intensity may blow up.

Simulating a Markov jump process is easy when intensities do not depend on time. In this case, given a state *x* at time *t*, we simply simulate reaction times $\tau _\ell \sim \textrm{Exp}(\lambda _\ell (x))$ for each reaction, set $\hat{\ell } = \mathop {\text {argmin}}\limits \nolimits _{\ell \in \mathcal {R}} \tau _\ell$ and move $t\leftarrow t+\tau _{\hat{\ell }}$ and $X(t+\tau _{\hat{\ell }})\leftarrow X(t)+\xi _{\hat{\ell }}$. When $\mathcal {R}$ contains many reactions, one could alternatively use Gillespie’s algorithm, see e.g. Gillespie (1976, 1977), which first samples the reaction time and subsequently the reaction that takes place at that time.

To extend this method to chemical reaction processes with time-dependent intensities, we have to sample reaction times satisfying (2.4). In general this is hard and therefore therefore we consider a Poisson thinning step. This gives Algorithm 1. The efficiency of Algorithm 1 will depend on whether sharp bounds $\bar{\lambda }_\ell$ can be derived.

**Algorithm 1:**
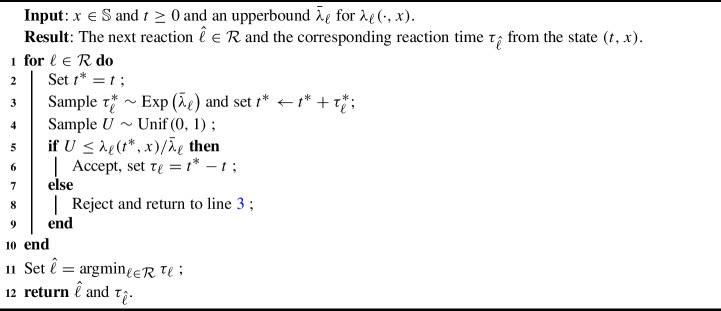
Next reaction method for time-inhomogeneous rates with a Poisson thinning step

### Simulation of the guided process for underlying processes with time-homogeneous intensities

In many applications, such as the examples in Section 2.3, the intensities $\lambda _\ell ,\, \ell \in \mathcal {R}$ of the underlying process only depend on the state *x* and don’t have a direct dependence on *t*. Here we consider this case and present a method for simulating the guided process in such a scenario. Note that if the underlying rates are time-dependent, but bounded, these upper bounds can easily be included in the thinning step.

#### Special case: guided process induced by $g_\varepsilon$

We consider the guided process with $\lambda _\ell ^{g_\varepsilon }=\alpha ^{g_\varepsilon }_\ell \lambda _\ell$, where $\alpha ^{g_\varepsilon }_\ell$ is defined in Eq. 4.8. As the map $t\mapsto \alpha _\ell ^{g_\varepsilon }(t,x)$ is monotone, given (*t*, *x*), an upper bound can be found at either (*t*, *x*) or (*T*, *x*). Unfortunately, the upper bound at *T* is typically far from sharp, hampering efficiency, especially when $\varepsilon$ is chosen to be small. To resolve this issue, we employ Algorithm 2. In lines 7–18, $\lambda _\ell ^{g_\varepsilon }(\cdot ,x)$ is increasing in time. Thus we can choose $\delta$ such that $t+\delta <T$ and use that, on the interval $[t, t+\delta ]$, $\lambda _\ell ^{g_\varepsilon }(\cdot , x)$ is upper bounded by $\lambda _\ell ^{g_\varepsilon }(t+\delta , x)$. Hence, on $[t,t+\delta ]$, we apply the thinning property for an inhomogeneous Poisson process with rate $\lambda _\ell ^{g_\varepsilon }(\cdot , x)$ and, if no reaction occurs in this interval, we move *t* to $t+\delta$. Typically, once $LX(t)=v$ for *t* near *T* the map $s\mapsto g(s,x+\xi _\ell )/g(s,x)$ will be decreasing for choices of *g* considered in this article. If at line 7$LX(t) \ne v$ *and* $T-(t+\delta )\le \varepsilon$ we reject the sampled path for specified small $\varepsilon >0$.

**Algorithm 2:**
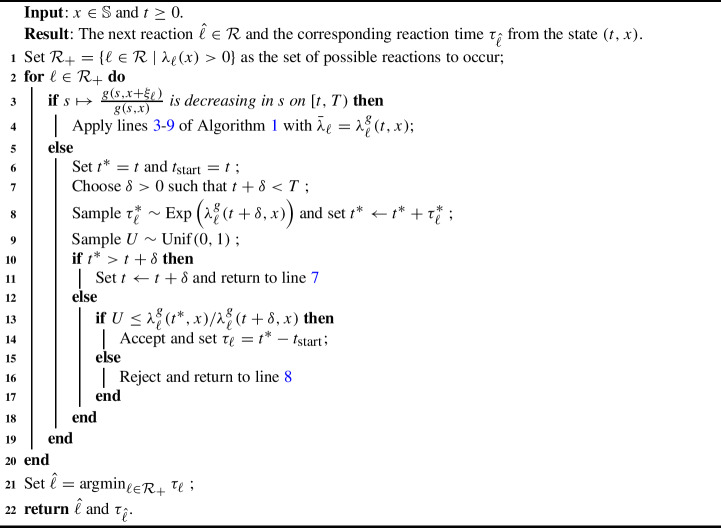
Next reaction method with a Poisson thinning step for guided rates where the original intensity is independent of time

#### Choice of $\delta$

We now explain that $\delta$ can be chosen such that a minimum acceptance rate is attained.

The acceptance rate of a proposed time $\tau _\ell$ is given by

6.1 $$\begin{aligned} \eta = \frac{\lambda _\ell ^{g_\varepsilon }(t+\tau _\ell ,x)}{\lambda _\ell ^{g_\varepsilon }(t+\delta ,x)} \end{aligned}$$

A direct computation yields that

6.2 $$\begin{aligned} \eta = \exp \left( \frac{d(v,L(x+\xi _\ell ))^2-d(v,Lx)^2}{2(T+\varepsilon -t-\delta )} - \frac{d(v,L(x+\xi _\ell ))^2-d(v,Lx)^2}{2(T+\varepsilon -t-\tau _\ell )}\right) , \end{aligned}$$

Upon solving (6.2) for $\delta$, we find that

$$\begin{aligned} \delta = T+\varepsilon -t-\left( \frac{2\log \eta }{d(v,L(x+\xi _\ell ))^2-d(v,Lx)^2} + \frac{1}{T+\varepsilon -t-\tau _\ell }\right) ^{-1} \end{aligned}$$

Now $\tau _\ell$ is not known when choosing $\delta$, but since $\tau _\ell \ge 0$, we obtain the desired acceptance rate $\eta$ by choosing

6.3 $$\begin{aligned} \delta \ge T+\varepsilon -t-\left( \frac{2\log \eta }{d(v,L(x+\xi _\ell ))^2-d(v,Lx)^2} + \frac{1}{T+\varepsilon -t}\right) ^{-1}. \end{aligned}$$

### Simulation studies

Julia written code for the simulation examples of this section is available in Corstanje (2023).

#### Death model and comparison to Golightly and Sherlock (2019)

We consider the pure death process of Example 2.2 and estimate the distribution of *X*(*T*) conditional on $X(0)=x_0$. For convenience, we denote by $g^v$ the guiding term *g* chosen for the conditioning $X(T)=v$. By Corollary 3.6, for any $v\in {\mathbb S}$

$$\begin{aligned} p(0,x_0;T,v) = g^v(0,x_0){\mathbb E}^{g^v} \left[ \frac{\Psi _T^{g^v}(X)}{g^v(T,X(T))}\textbf{1}_{\{X(T)=v\}} \right] \end{aligned}$$

Hence, for large *N*, we can estimate $p(0,x_0;T,v)$ by sampling $X_1,\dots , X_N$ from ${\mathbb P}^{g^v}$ using Algorithm 2 and computing

6.4 $$\begin{aligned} \hat{p}(v) = \frac{1}{N} \sum _{i=1}^N g^v(0,x_0)\frac{\Psi _T^{g^v}(X_i)}{g^v(T, X_i(T))}\textbf{1}_{\{X_i(T)=v\}} \end{aligned}$$

It is a well-known result that $X(T) \sim \textrm{Binom}\left( x_0, e^{-cT}\right)$, so we will use this mass function for comparison. In the following experiment, we use (6.4) to estimate the mass function of the $\textrm{Binom}\left( x_0, e^{-cT}\right)$-distribution with $x_0=50$, $T=1$ and $c=1/2$. We consider four choices for *g*:

- $g_F$ as in (4.1) with $C= 10^{-5}$ for $v<32$ and $C=0.3$ for $v\ge 32$ (we comment on this choice of *C* below).
- $g_{\textrm{LNA}}$ from the LNA method with restart as in (4.2), with $C = 10^{-5}$.
- $g_\varepsilon$ chosen using a scaled diffusion as in (4.4). We took $\varepsilon = 10^{-5}$. For a given value of *v*, the tuning parameter $a=\sigma ^2$ appearing in $g_\varepsilon$ was chosen as $2.5(x_0 -v)$. This was found to be roughly the optimum of the map $a\mapsto g^v(0,x_0){\mathbb E}^{g_{\varepsilon }}\Psi _T^{g^v}(X)\textbf{1}_{\{X(T)=v\}}/g^v(T,X(T))$, where the expectation was estimated using 100 forward simulated paths and *v* was taken to be the $1\%$, $50\%$ and $99\%$-quantiles of *X*(*T*).
- *g* chosen using the density of a reversed (decreasing) constant rate Poisson process with rate constant $\theta$. We took $\theta =c v$ (this choice ensures equivalence by Theorem 4.2 of Corstanje et al. (2023)).

For forward simulation under ${\mathbb P}^g$ we employed Algorithm 2 where for $g_\varepsilon$, $\delta$ was chosen according to (6.3). For the other choices of *g* such a closed form expression cannot be derived, and for $t<T$ we simply took $\delta = \frac{T-t}{2}$. This does not affect the validity of the algorithm, only the acceptance probability of a sampled reaction time.

**Fig. 3:**
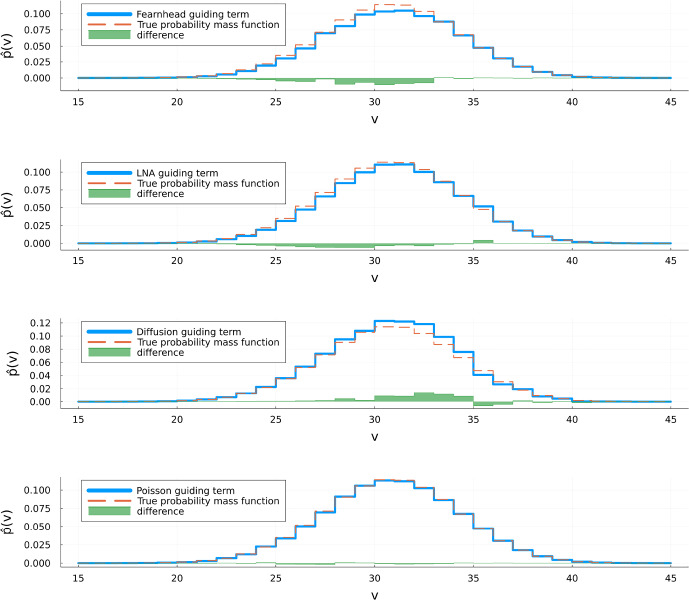
Estimates of the probability mass function of $X(T)\mid X(0)=x_0$ using the guiding functions *f* from the LNA method, a scaled diffusion and a Poisson process. The upper barplot is the true density. For each *v*, we estimated using (6.4) with $N = 15000$

**Fig. 4:**
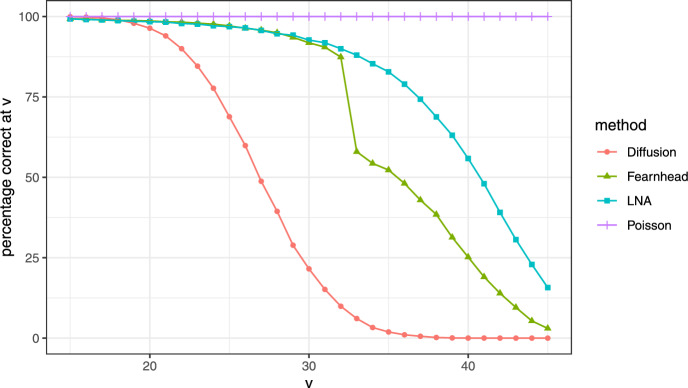
The percentage of paths ending in the point of conditioning (*v*) versus *v* for the four methods considered (with the same settings as in Fig. 3)

*Results:* We took Monte-Carlo sample size $N=15000$. The estimated probability mass functions are depicted in Fig. 3. This figure confirms that $\mathbb {P}_T^h\ll \mathbb {P}_T^g$ for all choices of *g* considered. The percentages of paths conditioned on $X(T)=v$ and actually ending in *v* are depicted in Fig. 4 for a range of values of *v*. We now comment on the choice of *C* for the Fearnhead guiding term. For $C=10^{-5}$ we noticed that conditioning on high values of *v* caused the guided process to jump very closely to *T* with high probability. This resulted in numerical instability when computing the integral appearing in $\Psi _T^g$. For that reason, we took a larger value of *C* in case *v* is large. A side effect of that is that there is less strong forcing to hit *v* at time *T* which explains the lower fractions of paths ending in *v* for $v\ge 32$.

We observe that the overall error of the Poisson guiding term is lowest and the diffusion guiding term performs particularly well for low values of *v* for which the process has to make a lot of jumps. However, when conditioning on high values, the amount of sample paths that end up in the correct state is low, leading to a larger variance of $\hat{p}(v)$ defined in Eq. 6.4. The performance of the Poisson guiding term is explained due to the typically lower guiding term, which is exactly 0 when the conditioning is reached at a time prior to *T*. This property is not shared by the other choices considered.

In Table 1 we report the mean squared errors $\frac{1}{\#v}\sum _v (q(v)-\hat{p}(v))^2$, with *q* denoting the probability mass function of the $\textrm{Binom}(x_0,e^{-cT})$-distribution.

**Table 1:** Mean squared errors of the estimates from Fig. 3 for each of the methods

Method	Mean Squared Error
Fearnhead	$1.5\cdot 10^{-5}$
LNA (with restart)	$6.5\cdot 10^{-6}$
Diffusion guiding term	$2.1\cdot 10^{-5}$
Poisson guiding term	$4.0\cdot 10^{-7}$

#### Gene transcription and translation

**Fig. 5:**
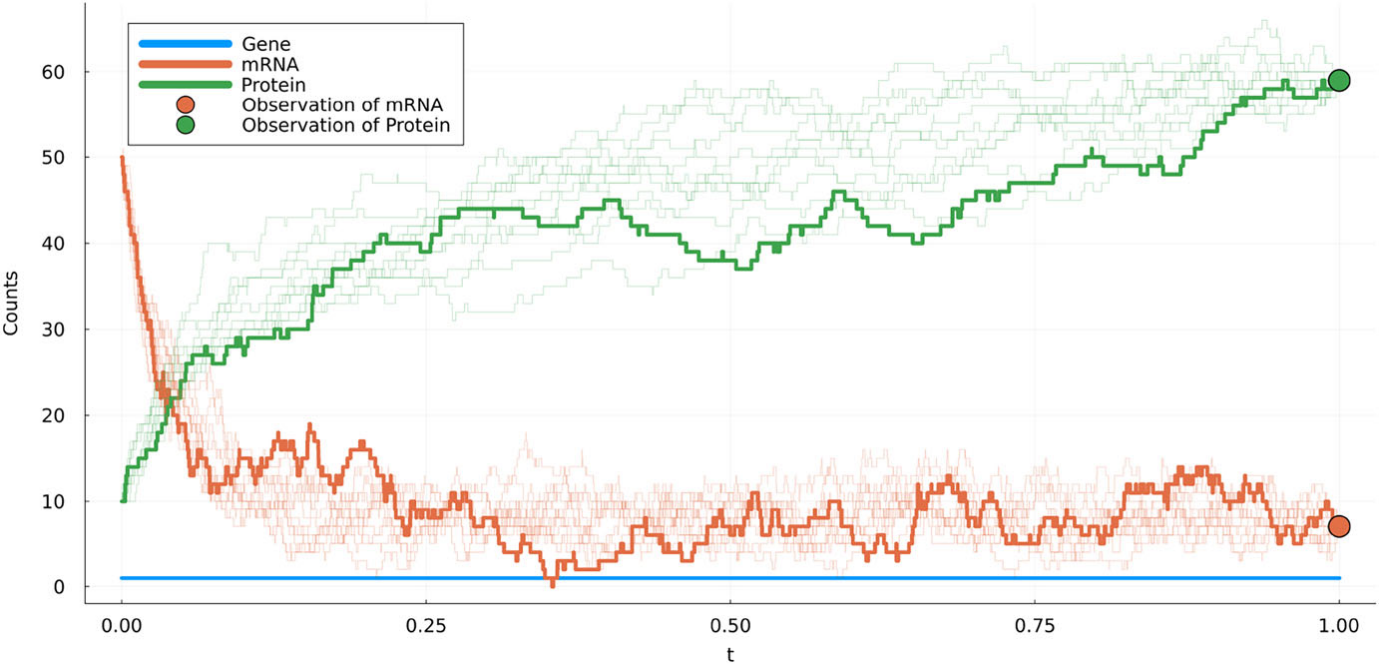
Realization of a guided process starting from $x_0=(1,50,10)$ conditioned to be at (1, 10, 50) at time $T=1$ of the *GTT*-model from Example 2.3 with $\kappa _1 = 100$, $\kappa _2 = 10$, $d_M = 25$ and $d_P=1$. The thick line is the original (unconditioned) process

We consider the *GTT*-model presented in Example 2.3. Figure 5 contains realizations of the process *X* under ${\mathbb P}^{g}$ conditioned to hit (1, 11, 56) at time $T=1$. We used guiding induced by $g_\varepsilon$ with $\varepsilon =10^{-5}$ and $a = a_{\textrm{CLE}}(0,x_0)$. The plot shows 10 sampled trajectories. Out of 1000 more trajectories sampled, all of these end in the correct point $X(T)=(1,11,56)$.

**Fig. 6:**
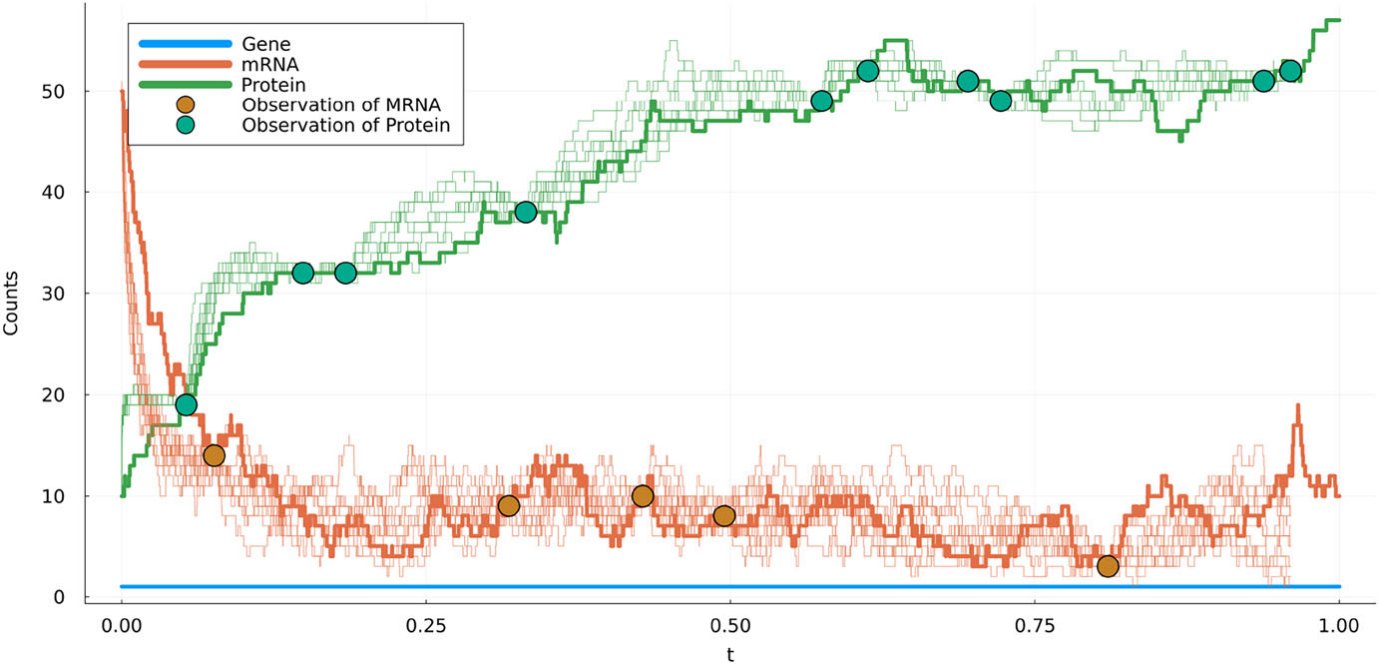
Realization of a guided process through 15 arbitrary observations, with the same settings as in Fig. 5

Next, from a simulated forward path we chose 15 times at random and saved the values of randomly chosen components of the process. Taking these values as observations we show in Fig. 6 multiple realisations of the guided process. Out of 1000 simulated trajectories we found that all of those passed through all partial observations.

#### Enzyme kinetics

As described in Example 2.4, this is a very interesting example as it contains a monotone component as well as absorbing states, where no more reactions can occur. For example, the state $X(t) = (0,20,0,32)$ can be reached with a sequence of reactions from $x_0$, but no further reactions are possible in this state. We use this example to compare various guiding terms. We choose the starting point $x_0 = (12,10,10,10)$ and parameters $(\kappa _1, \kappa _2, \kappa _3) = (5,5,3)$. We consider three scenarios in which the LNA method with restart, the guiding term obtained from a scaled diffusion presented in Sect. 6.2.3 and the guiding term in which one of the components is replaced by a Poisson guiding term as presented in Sect. 4.3.

- **Scenario A: ** The process is conditioned to be at $x_{T,0.01} = (0,15,5,27)$ at time $T=1$. 27 is the $1\%$ quantile of $X_4(T)$, determined through forward simulation.
- **Scenario B: ** The process is conditioned to be at $x_{T,0.5} = (0,19,1,31)$ at time $T=1$. 31 is the $50\%$ quantile of $X_4(T)$, determined through forward simulation.
- **Scenario C: ** The process is conditioned to be at $x_{T,0.99} = (0,20,0,32)$ at time $T=1$. 32 is the $99\%$ quantile of $X_4(T)$, determined through forward simulation and $x_{T,0.99}$ is also an absorbing state of the process.

**Fig. 7:**
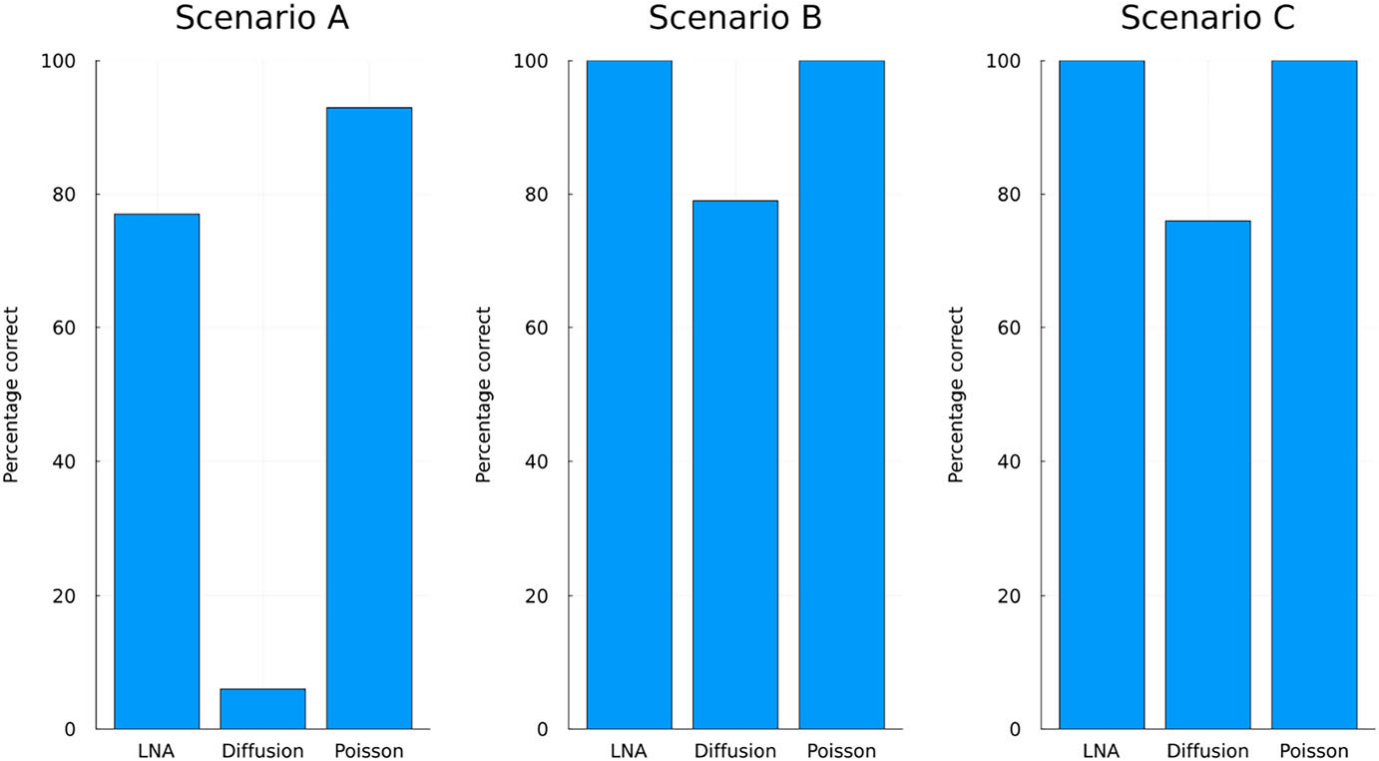
Percentages out of 100 trajectories that satisfy the conditionings set in scenario’s **A**, **B** and **C**

First we simulate 100 trajectories of each process and check the percentage of paths that satisfy $X(T) = x_{T,q}$ for $q=0.01,0.5,0.99$. We used the same scheme for simulating the guided process as for the death process. For the diffusion guiding term, we took $a = a_{\textrm{CLE}}(0,x_0)$. For the Poisson guiding term, the auxiliary process from which *g* is obtained contains a scaled diffusion in the 3 components and a Poisson processes in the third component. The diffusion is scaled by a matrix *a*, in which we used the first three rows and the first three columns of $a_{\textrm{CLE}}(0,x_0)$. The intensity of the Poisson component was taken as $\lambda _3(0,x_0)$, which is a lower bound of the reaction intensity for the reaction that induces the monotone component. The results are summarised in Fig. 7.

Similar to the death process in Fig. 3, we see that the diffusion guiding term and the LNA guiding term struggle when the monotone component is conditioned not to make many jumps.

**Fig. 8:**
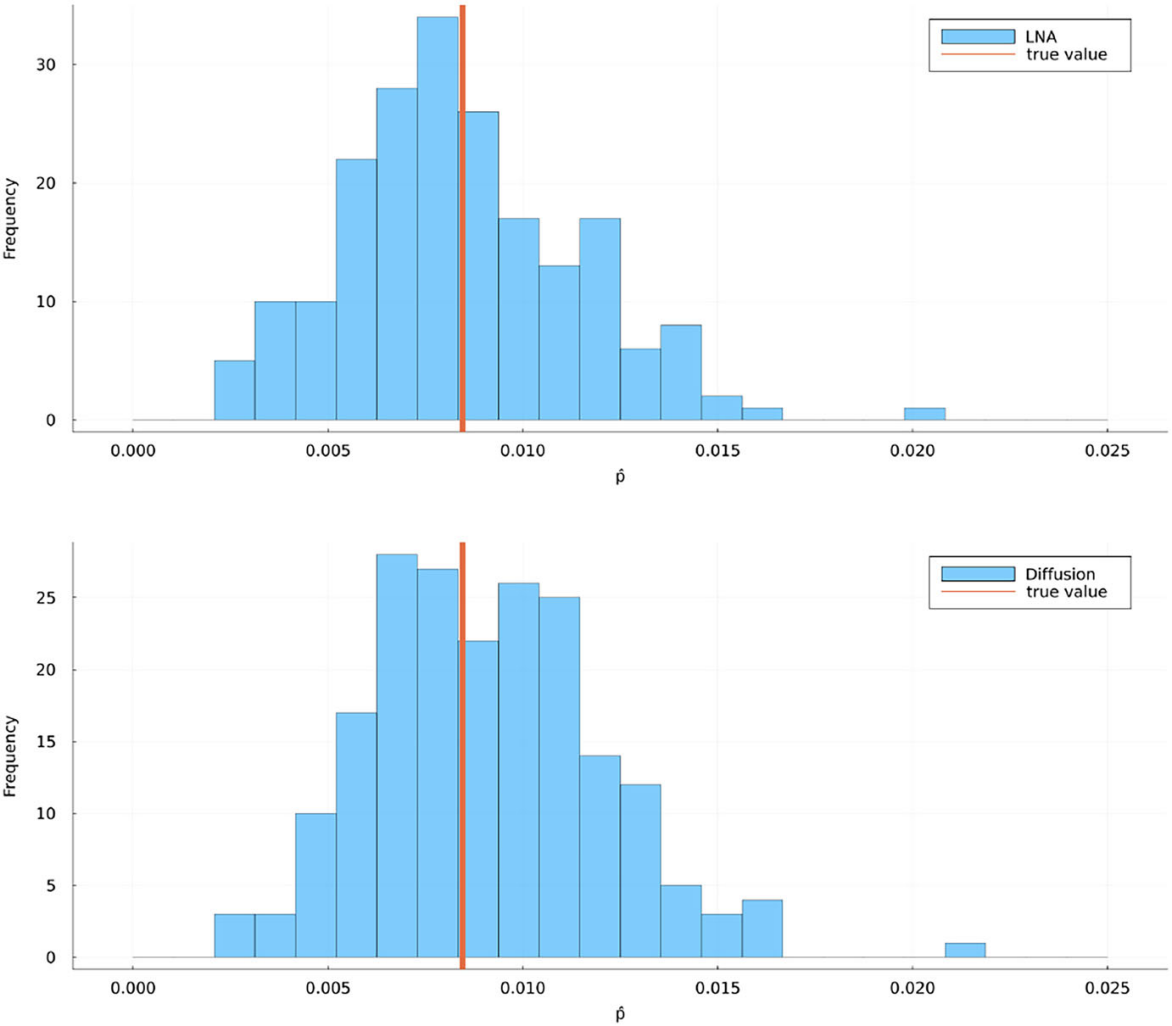
Histograms of 200 estimates for $p(0,x_0;T,v)$ in scenario A using the LNA method without restart (top) and and the diffusion method (bottom). The true value was estimated using 10,000 forward simulations

**Fig. 9:**
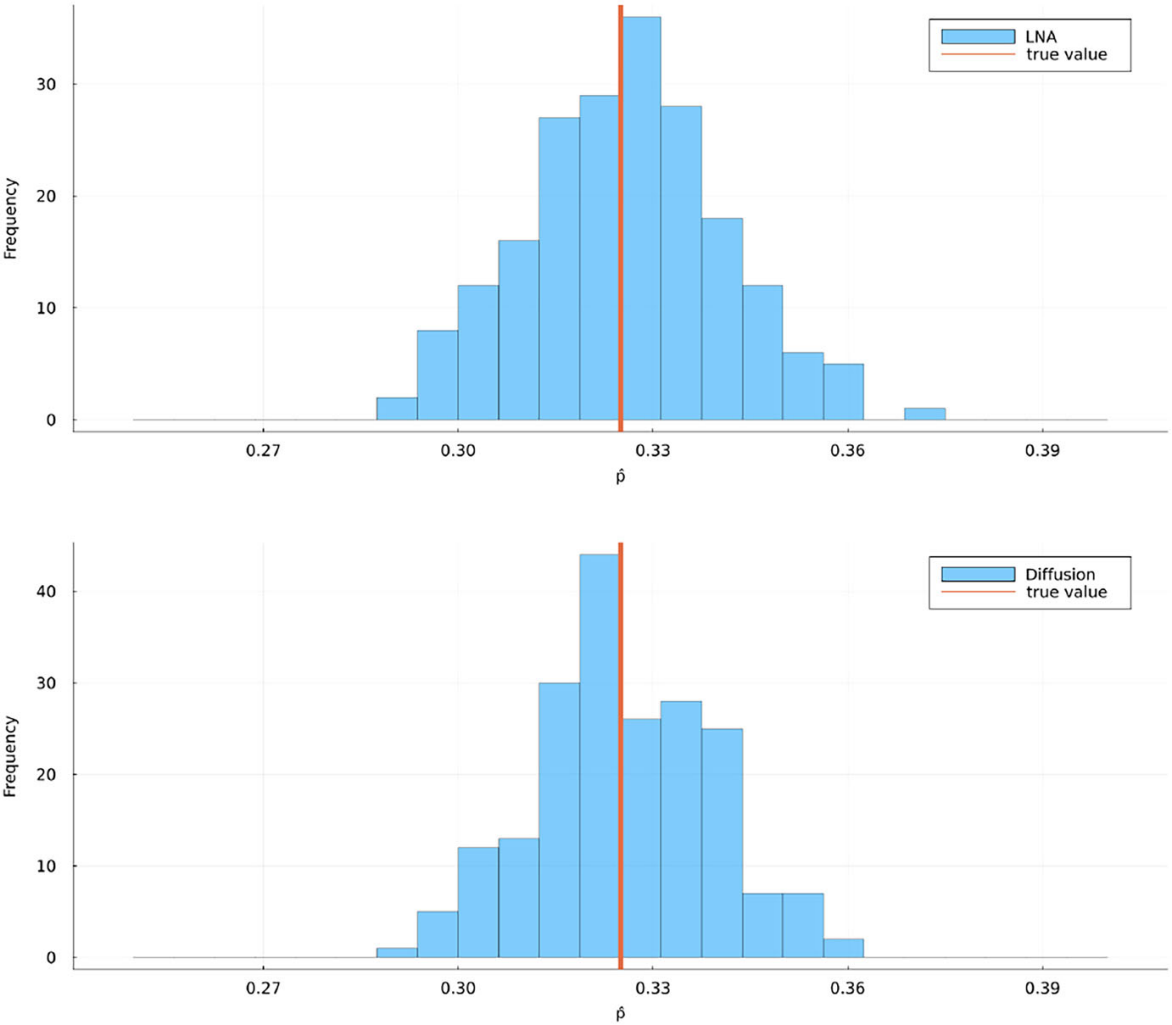
Histograms of 200 estimates for $p(0,x_0;T,v)$ in scenario B using the LNA method without restart (top) and and the diffusion method (bottom). The true value was estimated using 10,000 forward simulations

**Fig. 10:**
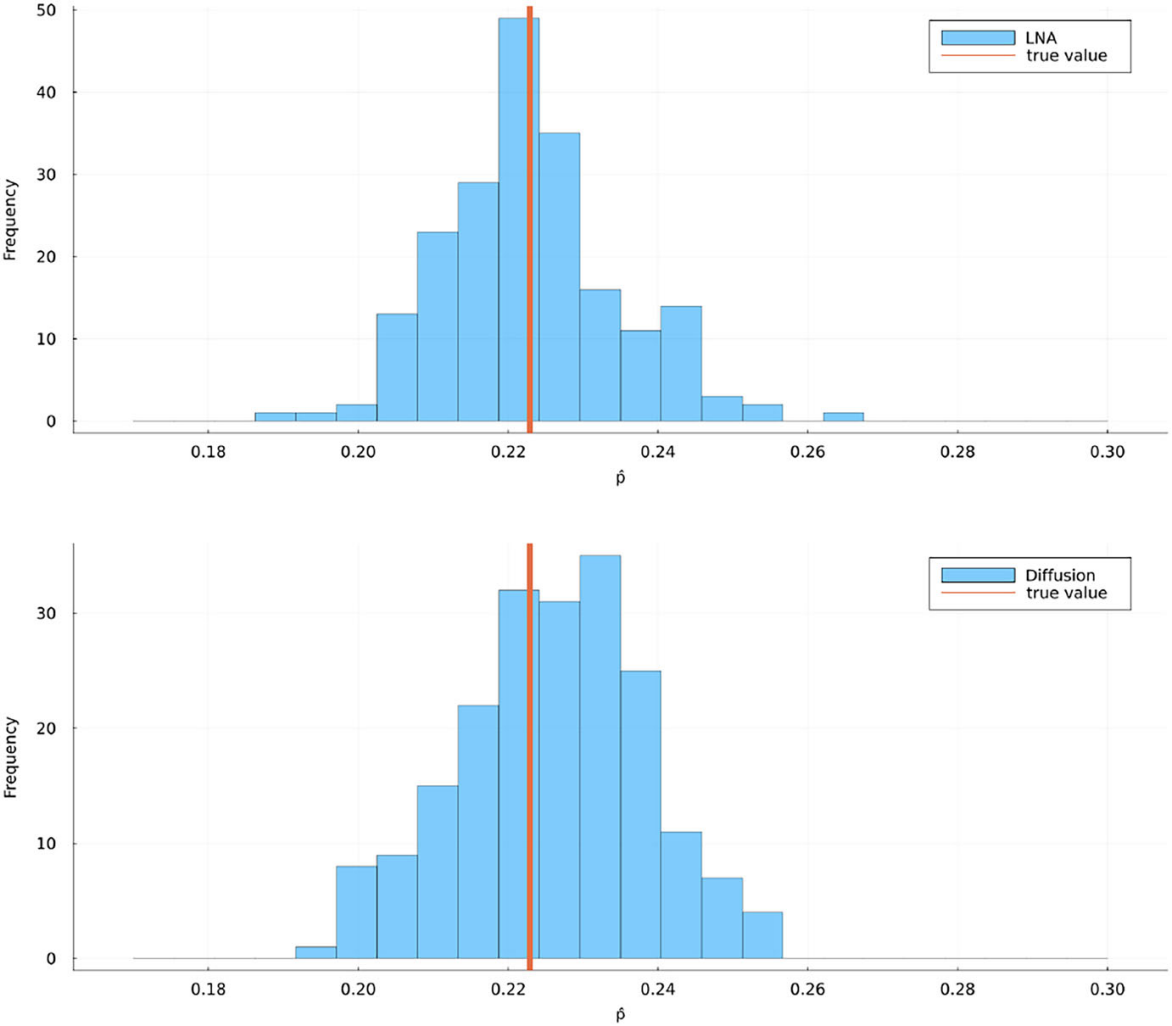
Histograms of 200 estimates for $p(0,x_0;T,v)$ in scenario C using the LNA method without restart (top) and and the diffusion method (bottom). The true value was estimated using 10,000 forward simulations

We used the same method as for the death processes to estimate $p(0,x_0; T, v)$ for scenario’s A, B and C. In each scenario, we computed $\hat{p}$ through (6.4). In each scenario, we make 200 estimates for $p(0,x_0; T, v)$ by computing $\hat{p}$ with $N=1000$ processes and we compared the LNA method (without restart cf. Section 4.3 of Golightly and Sherlock (2019)) with the diffusion guiding term with $\varepsilon = 10^{-5}$ and $a = 100a_{\textrm{CLE}}(T,x_{T,q})$ for $q=0.01,0.5,0.99$. For the LNA method we assumed extrinsic noise $C=2000I$, $C=500I$ and $C=200I$ for scenario’s A, B and C, respectively. Histograms of the estimates are given in Figs. 8, 9 and 10. The MSEs of the estimates are summarised in Table 2.

**Table 2:** Mean Squared Error (MSE) of the estimates from Figs. 8, 9 and 10 for each of the methods

Method	MSE scenario A	MSE scenario B	MSE scenario C
LNA (without restart)	$8.8\cdot 10^{-6}$	$2.3\cdot 10^{-3}$	$1.3 \cdot 10^{-3}$
Diffusion guiding term	$9.0\cdot 10^{-6}$	$1.8 \cdot 10^{-3}$	$1.6 \cdot 10^{-3}$

## Discussion

In this paper we have provided sufficient conditions for constructing valid guided processes, where “valid” refers to the law of the true conditioned process being absolutely continuous with respect to the law of the guided process induced by *g* with the laws as defined in Sect. 3. We have presented various choices of *g* and conclude that among those there is no best choice in terms of closeness to the true conditioned process and computational cost. When used within a sequential Monte Carlo or Markov chain Monte Carlo algorithm, a mixture of proposals may be beneficial. The inherent discreteness of chemical reaction processes makes it a hard problem, but it works to our advantage in the sense that guided processes can be constructed on [0, *T*] where *g*(*T*, *x*) is well defined. This is accomplished by using a guiding term derived from conditioning a diffusion process that is observed with (small) extrinsic noise. The guiding term defined in Sect. 4.3 has the additional advantage that monotonicity can be exploited for simulating paths of the guided process efficiently.

In the analogous problem of continuous-discrete smoothing for diffusion processes (see e.g. Mider et al. (2021), Beskos et al. (2008), Golightly and Wilkinson (2008)) there exists a simple random-walk like MCMC-sampler on path space to update guided processes: the preconditioned Crank-Nicolson scheme. Unfortunately, we are not aware of a similar construct for chemical reaction processes.

## Appendix A Proof of Theorem 4.2

The proof relies on the existence of a chain of reactions that result in the conditioning $LX(T)=v$. We formalize this in Definition A.1.

### Definition A.1

For $x,y\in {\mathbb S}$, we say that *y* can be reached from *x* within a time interval $(a,b)\subseteq (0,T)$, denoted $x\hookrightarrow y$ in (*a*, *b*), if there is a finite collection $\{ \ell _1,\dots ,\ell _I\}\subseteq \mathcal {R}$ of reactions and a partition $U_1,\dots , U_I$ of disjoint intervals for (*a*, *b*) such that $y=x +\sum _{i=1}^I \xi _{\ell _i}$ and for all the set *i*, $\textrm{supp}\, \lambda _{\ell _i}\left( x+\sum _{j<i}\xi _{\ell _j}\right) \cap U_i$ is of positive Lebesgue measure.

Since we consider a process conditioned on $LX(T)=v$, we can assume there exists an $x_T\in {\mathbb S}$ such that $Lx_T=v$ and $x_0\hookrightarrow x_T$ in (0, *T*).

### Proof of Theorem 4.2

Notice that, given any $t<T$,

$$\begin{aligned} {\mathbb P}^{g}\left( X(s)=x_T\text { for all }s\in [t,T]\mid X(t)=x_T\right) = \exp \left( -\int _t^T\sum _{\ell \in \mathcal {R}} \lambda _{\ell }^{g}(s,x_T)\,\textrm{d}s \right) . \end{aligned}$$

Now it can be shown that $\sum _{\ell \in \mathcal {R}} \lambda _\ell ^{g}$ is bounded through Lemma C.1 and Assumption Assumption (2.1c) that the right hand side is strictly positive and hence, it suffices to show that ${\mathbb P}^{g}\left( \exists t<T: X(t)=x_T\right) >0$. In the remainder of this proof, we show this by showing that the probability that the chain of reactions described in Definition A.1 connection $x_0$ to $x_T$ occurs is positive.

Let $\ell _1,\dots ,\ell _I$ be the collection of reactions described in Definition A.1 and let $U_1,\dots ,U_I$ be the respective partition of (0, *T*). For the first reaction to occur, we denote by $T_\ell ^{(1)}$ the reaction time for each reaction $\ell \in \mathcal {R}$ and let $L_1$ be the first reaction to occur. That is,

$$\begin{aligned} L_1 = \mathop {\text {argmin}}\limits _{\ell \in \mathcal {R}} T_\ell ^{(1)},\qquad \text {where}\qquad {\mathbb P}^{g}\left( T_\ell ^{(1)}>t\right) = \exp \left( -\int _0^t \lambda _\ell ^{g}(s,x_0)\,\textrm{d}s\right) . \end{aligned}$$

Note that,

$$\begin{aligned} \left\{ T_{\ell _1}^{(1)} \in U_1,\, T_\ell ^{(1)}>\sup \, U_1 \text { for all }\ell \in \mathcal {R}\setminus \{\ell _1\}\right\} \subseteq \left\{ T_{\ell _1}^{(1)}\in U_1,\, L_1=\ell _1 \right\} \end{aligned}$$

and thus

$$\begin{aligned} &  {\mathbb P}^{g}\left( T_{\ell _1}^{(1)} \in U_1,\, L_1=\ell _1\right) \\  &  \quad \ge \left( 1- \exp \left( -\int _{U_1}\lambda ^{g}_{\ell _1}(s,x_0)\,\textrm{d}s \right) \right) \exp \left( -\int _{U_1} \sum _{\ell \in \mathcal {R}\setminus \{\ell _1\}} \lambda _\ell ^{g}(s,x_0)\,\textrm{d}s\right) . \end{aligned}$$

Since $\lambda _{\ell _1}^{g}(\cdot ,x_0)$ has support in $U_1$, the first term is nonzero, while the second term is nonzero by Assumption (2.1c).

For the second reaction, we set

$$\begin{aligned} L_2 = \mathop {\text {argmin}}\limits _{\ell \in \mathcal {R}} T_\ell ^{(2)} \qquad \text {where}\qquad {\mathbb P}^{g} \left( T_\ell ^{(2)}>t \mid L_1 \right) = \exp \left( -\int _{T_{L_1}^{(1)}}^t \lambda _{\ell }^{g}(s, x_0+\xi _{L_1}) \,\textrm{d}s\right) . \end{aligned}$$

Through similar reasoning, we deduce that

$$\begin{aligned} \begin{aligned}&{\mathbb P}^{g} \left( L_2 = \ell _2,\, T_{\ell _2}^{(2)} \in U_2,\, L_1 = \ell _1,\, T_{\ell _1}^{(1)} \in U_1\right) \\&\quad = {\mathbb P}^{g}\left( L_2 = \ell _2,\, T_{\ell _2}^{(2)} \in U_2 \mid L_1 = \ell _1,\, T_{\ell _1}^{(1)} \in U_1\right) {\mathbb P}^{g}\left( L_1 = \ell _1,\, T_{\ell _1}^{(1)} \in U_1)\right) \\&\quad \ge \left( 1- \exp \left( -\int _{U_2}\lambda _{\ell _2}^{g}\left( s, x_0 + \xi _{\ell _1}\right) \,\textrm{d}s \right) \right) \exp \left( -\int _{U_2} \sum _{\ell \in \mathcal {R}\setminus \{\ell _2\}} \lambda _{\ell }^{g} (s, x_0+\xi _{\ell _1}) \,\textrm{d}s \right) \\&\quad \quad \times \left( 1- \exp \left( -\int _{U_1}\lambda ^{g}_{\ell _1}(s,x_0)\,\textrm{d}s \right) \right) \exp \left( -\int _{U_1} \sum _{\ell \in \mathcal {R}\setminus \{\ell _1\}} \lambda _\ell ^{g}(s,x_0)\,\textrm{d}s\right) . \end{aligned} \end{aligned}$$

Upon iteratively repeating this process, we find

$$\begin{aligned} &  {\mathbb P}^{g}\left( L_i = \ell _i,\, T_{\ell _i}^{(i)} \in U_i,\, i=1,\dots ,I\right) \ge \\ &  \prod _{i=1}^I \left( 1- \exp \left( -\int _{U_i}\lambda _{\ell _i}^{g}\left( s, x_0+\sum _{j<i}\xi _{\ell _j}\right) \,\textrm{d}s\right) \right) \\ &  \times \exp \left( -\int _{U_i}\sum _{\ell \in \mathcal {R}\setminus \{\ell _i\}}\lambda _{\ell _i}^{g}\left( s,x+\sum _{j<i}\xi _{\ell _j}\right) \,\textrm{d}s\right) . \end{aligned}$$

The right hand size is strictly positive as a finite product of nonzero terms. Therefore ${\mathbb P}^{g}\left( \exists t\le T: X(t)=x_T\right) >0$, which finishes the proof. $\square$

## Appendix B Proofs for Sect. 5

### Theorem B.1

(Theorem 3.3 of Corstanje et al. (2023)]) Suppose that there exist a family of $\mathcal {F}_t$-measurable events $\{A_j[X^t] \}_j$ for each $t\in [0,t_n)$ so that the following assumptions hold.

- For all *j* $s\le t$, $A_j[X^t]\subseteq A_{j+1}[X^t]$ and $A_j[X^t]\subseteq A_j[X^s]$.
- For all *k* and $s\in [0,t_k)$ and $x\in {\mathbb S}$,
  $$\begin{aligned} \lim _{t\uparrow t_k} {\mathbb E}\left[ g(t,X(t))\mid X(s)=x\right] = {\mathbb E}\left[ g(t_k, X(t_k))\textbf{1}\{L_kX(t_k)=v_k\}\right] , \end{aligned}$$
  Moreover, ${\mathbb E}\left[ g(t,X(t))\mid X(s)\right]$ is ${\mathbb P}$-almost surely bounded for all *t*.
- For all $$k=1,\dots ,n$$,
  $$\begin{aligned} \lim _{j\rightarrow \infty } \lim _{t\uparrow t_k} {\mathbb E}^{h}_t \left[ \frac{g(t, X(t))}{h(t, X(t))} \textbf{1}_{A_j[X^t]} \right] = \frac{{\mathbb E}\left[ g(t_k, X(t_k))\textbf{1}\{L_kX(t_k)=v_k\}\textbf{1}_{A[X^{t_k}]}\right] }{h(0,x_0)},\end{aligned}$$
  where $$A_j[X^{t_k}] = \cap _{t\in [0,t_k)} A_j[X^t]$$, $$A[X^t] = \bigcup _j A_j[X^t]$$ and $$A[X^{t_k}]=\cap _{t\in [0,t_k)} A[X^t]$$.
- For all fixed *j*, $\Psi _t^g(X)\textbf{1}_{A_j[X^t]}$ is ${\mathbb P}^{g}$-almost surely uniformly bounded in *t*.

Then for any bounded measurable function *f*,

B.1 $$\begin{aligned} {\mathbb E}\left[ f(X)\frac{ {\mathbb E}\left[ g(t_k,X(t_k))\textbf{1}\{L_kX(t_k)=v_k\}\right] }{h(0,x_0)}\textbf{1}_{A[X^{t_k}]} \right] = {\mathbb E}^{g}\left[ f(X) \frac{g(0,x_0)}{h(0,x_0)}\Psi _{t_k}^g(X)\textbf{1}_{A[X^{t_k}]} \right] \end{aligned}$$

for $k=1,\dots ,n$. In particular, for $k=n$,

B.2 $$\begin{aligned} {\mathbb E}^{h} \left[ f(X)\textbf{1}_{A[X^{t_n}]} \right] = {\mathbb E}^{g}\left[ f(X) \frac{g(0,x_0)}{h(0,x_0)}\Psi _{t_n}^g(X)\textbf{1}_{A[X^{t_n}]} \right] . \end{aligned}$$

### Proof

The proof of Theorem 3.3 of Corstanje et al. (2023) can be followed with the new limits in (B.1b) and (B.1c) for each of the limits $t\uparrow t_k$. $\square$

### Proof of Theorem 5.1

The proof utilizes Theorem B.1 with

B.3 $$\begin{aligned} V(t,x) = \sum _{\ell \in \mathcal {R}} \lambda _\ell ^{g}(t,x) \qquad \text {and}\qquad A_j[X^t] = \left\{ \sup _{0\le s<t} V(s,X(s))\le j \right\} . \end{aligned}$$

Then (B.1a) is satisfied by construction. Lemmas B.3-B.5 prove the remaining conditions. $\square$

### Lemma B.2

The map *g*, defined in (5.1), is such that $g\in \mathcal {G}$ and for all $k=1,\dots ,n$ and $x\in {\mathbb S}$,

B.4 $$\begin{aligned} \lim _{t\uparrow t_k} g(t,x) = {\left\{ \begin{array}{ll} g(t_k,x) & \text {if }L_kx=v_k \\ 0 & \text {otherwise} \end{array}\right. }. \end{aligned}$$

and $\partial _{t}\log g(t,x)\le 0$ for all $(t,x)\in (0,t_n)\times {\mathbb S}$.

### Proof

Through a direct computation using the Schur complement, it can be derived that as $t\uparrow t_k$,

B.5 $$\begin{aligned} (v(t)-L(t)x)^{\prime }M(t)(v(t)-L(t)x) = \frac{d_k(v_k,L_kx)^2}{(t_k-t)} + o(t_k-t), \end{aligned}$$

where $d_k$ is the metric on ${\mathbb R}^{m_k}$ defined as

B.6 $$\begin{aligned} d_k(x,y) = \sqrt{(y-x)^{\prime }\left( L_ka_k L_k^{\prime }\right) ^{-1}(y-x)}, \qquad x,y\in {\mathbb R}^{m_k}. \end{aligned}$$

which yields (B.4). Since *v* and *L* are piecewise constant and $${\frac{\,\textrm{d}M}{\,\textrm{d}t}} = M(t) \begin{pmatrix} L_ia_k L_j^{\prime }\end{pmatrix}_{i,j=k}^n M(t)$$ is positive semidefinite, $\partial _t\log g(t,x)\le 0$. $\square$

### Lemma B.3

For all $k=1,\dots , n$, $s\in [0,t_n)$ and $x\in {\mathbb S}$,

$$\begin{aligned} \lim _{t\uparrow t_k}{\mathbb E}\left[ g(t,X(t)) \mid X(s)=x\right] = {\mathbb E}\left[ g(t_k, X(t_k))\textbf{1}\{ L_kX(t_k)=v_k \}\mid X(s)=x\right] . \end{aligned}$$

### Proof

By dominated convergence and Lemma B.2

$$\begin{aligned} \begin{aligned}&\lim _{t\uparrow t_k} {\mathbb E}\left[ g(t,X(t))\mid X(s)=x \right] \\&= \lim _{t\uparrow t_k} \sum _{y\in {\mathbb S}} g(t,y)p(s,x;t,y)\\&= \sum _{y\in {\mathbb S}} g(t_k,y)\textbf{1}\{ L_ky=v_k \}p(s,x;t_k,y)\\&= {\mathbb E}\left[ g(t_k,X(t_k))\textbf{1}\{L_kX(t_k)=v_k\}\mid X(s)=x\right] . \end{aligned} \end{aligned}$$

$$\square $$

### Lemma B.4

For all $$k=1,\dots ,n$$,

$$\begin{aligned} \lim _{j\rightarrow \infty } \lim _{t\uparrow t_k} {\mathbb E}^{h} \left[ \frac{g(t,X(t))}{h(t,X(t))}\textbf{1}_{A_j[X^t]} \right] = \frac{ {\mathbb E}\left[ g(t_k,X(t_k))\textbf{1}\{ L_kX(t_k)=v_k \}\textbf{1}_{A[X^{t_k}]} \right] }{h(0,x_0)}. \end{aligned}$$

### Proof

Upon defining $Z(t) = g(t,X(t))$, it follows from dominated convergence and Lemma B.2 that for any bounded continuous function *f*,

$$\begin{aligned} \begin{aligned} \lim _{t\uparrow t_k} {\mathbb E} f(Z(t))&= \sum _{x\in {\mathbb S}}\lim _{t\uparrow t_k} f\left( g(t,x)\right) p(0,x_0;t,x) \\&= \sum _{x\in {\mathbb S}} f\left( g(t_k,x)\textbf{1}\{ L_k x =v_k \}\right) p(0,x_0;t_k,x) \\&= {\mathbb E}f\left( g(t_k,X(t_k))\textbf{1}\{ L_k X(t_k) =v_k \} \right) . \end{aligned} \end{aligned}$$

Hence,

B.7 $$\begin{aligned} Z(t)\rightsquigarrow g(t_k,X(t_k))\textbf{1}\{ L_k X(t_k) =v_k \}\text { as }t\uparrow t_k. \end{aligned}$$

Now

$$\begin{aligned} {\mathbb E}\left[ Z(t)\textbf{1}_{A_j[X^t]} \right] = {\mathbb E}\left[ Z(t)\textbf{1}_{A_j[X^{t_k}]}\right] +{\mathbb E}\left[ Z(t)\textbf{1}_{A_j[X^t]\setminus A_j[X^{t_k}]}\right] \end{aligned}$$

By (B.7), the first term tends to ${\mathbb E}\left[ g(t_k,X(t_k))\textbf{1}\{ L_k X(t_k) =v_k \}\textbf{1}_{A_j[X^{t_k}]} \right]$ as $t\uparrow t_k$. Moreover, $\textbf{1}_{A_j[X^t]\setminus A_j[X^{t_k}]} \downarrow 0$ as $t\uparrow t_k$ and thus the second term tends to 0 by Slutsky’s theorem. Hence, by (3.5)

$$\begin{aligned} \begin{aligned} \lim _{j\rightarrow \infty } \lim _{t\uparrow t_k} {\mathbb E}^{h} \left[ \frac{Z(t)}{h(t,X(t))} \textbf{1}_{A_j[X^t]}\right]&= \frac{1}{h(0,x_0)}\lim _{j\rightarrow \infty }\lim _{t\uparrow t_k} {\mathbb E}\left[ Z(t)\textbf{1}_{A_j[X^t]}\right] \\&= \frac{1}{h(0,x_0)}\lim _{j\rightarrow \infty }{\mathbb E}\left[ g(t_k,X(t_k))\textbf{1}\{ L_k X(t_k) =v_k \} \textbf{1}_{A_j[X^{t_k}]} \right] \\&= \frac{ {\mathbb E}\left[ g(t_k,X(t_k))\textbf{1}\{ L_kX(t_k)=v_k \}\textbf{1}_{A[X^{t_k}]} \right] }{h(0,x_0)}. \end{aligned} \end{aligned}$$

$$\square $$

### Lemma B.5

For all *j*,

$$\begin{aligned} \sup _{t<t_n} \exp \left( \int _0^t \frac{\mathcal {A}g}{g}(s,X(s))\,\textrm{d}s \right) \textbf{1}_{A_j[X^t]} \le \exp (jt_n). \end{aligned}$$

### Proof

Since $$\mathcal {A}=\partial _t+\mathcal {L}$$,

$$\begin{aligned} \frac{\mathcal {A}g}{g}(s,x) = \partial _s\log g(s,x) + \frac{\mathcal {L}g}{g}(s,x). \end{aligned}$$

Note that

$$\begin{aligned} \frac{\mathcal {L}g}{g}(s,x) = \sum _{\ell \in \mathcal {R}} \frac{\lambda _\ell (s,x)\left( g(s,x+\xi _\ell )-g(s,x)\right) }{g(s,x)} = V(s,x)-\sum _{\ell \in \mathcal {R}}\lambda _\ell (s,x) \le V(s,x). \end{aligned}$$

By Lemma B.2, $\partial _t\log g \le 0$ and hence, for all $t<t_n$ and on $A_j[X^t]$,

$$\begin{aligned} \int _0^t \frac{\mathcal {A}g}{g}(s,X(s))\,\textrm{d}s = \int _0^t \partial _s\log g(s,X(s))\,\textrm{d}s + \int _0^t \frac{\mathcal {L}g}{g}(s,X(s))\le jt \le jt_n. \end{aligned}$$

$$\square $$

### Proof of Proposition 5.2

By (B.5),

B.8 $$\begin{aligned} \lim _{t\uparrow t_k} \frac{g(t,x+\xi _\ell )}{g(t,x)} = {\left\{ \begin{array}{ll} \frac{g(t_k,x+\xi _\ell )}{g(t_k,x)} & \text {if }L_kx=L_k(x+\xi _\ell )=v_k \\ 0 & \text {if }d_k(v_k,L_kx)>d_k(v_k,L_k(x+\xi _\ell ) \\ \infty & \text {if }d_k(v_k,L_kx) < d_k(v_k,L_k(x+\xi _\ell ) \end{array}\right. }. \end{aligned}$$

And therefore

B.9 $$\begin{aligned} \lim _{t\uparrow t_k} \lambda _\ell ^{g}(t,x) = {\left\{ \begin{array}{ll} \lambda _\ell (t_k,x)\frac{g(t_k,x+\xi _\ell )}{g(t_k,x)} & \text {if }L_kx=L_k(x+\xi _\ell )=v_k \\ 0 & \text {if }d_k(v_k,L_k(x+\xi _\ell )) > d_k(v_k,L_kx )\\ \infty & \text {if }d_k(v_k,L_k(x+\xi _\ell )) < d_k(v_k,L_kx ) \end{array}\right. }. \end{aligned}$$

Now, for $t\in [t_{k-1},t_k)$, define $B_t^k = \{ L_kX(s)=v_k\text { for all }s\in [t,t_k]\}$. Then it follows that for all *k* and $t\in [t_{k-1},t_k)$,

$$\begin{aligned} B_t^k \subseteq \left\{ \sup _{t_{k-1}\le s<t_k}\, \sum _{\ell \in \mathcal {R}}\lambda _\ell ^{g}(s,X(s)) <\infty \right\} . \end{aligned}$$

Hence

$$\begin{aligned} A_n = \bigcup _{k=1}^n \bigcap _{t_{k-1}\le t<t_k} B_t^k \subseteq \bigcup _{k=1}^n \left\{ \sup _{t_{k-1}\le s<t_k}\, \sum _{\ell \in \mathcal {R}}\lambda _\ell ^{g}(s,X(s)) <\infty \right\} = A[X^{t_n}]. \end{aligned}$$

$$\square $$

### Proof of Theorem 5.5

Observe that the result follows immediately if we show that for $k=1,\dots ,n$, ${\mathbb P}^g(L_k(X(t_k)=v_k) >0$. Now note The probability distribution of the next reaction time $\tau$ satisfies, for $t_{k-1}<s<t<t_k$ and $x\in {\mathbb S}$,

$$\begin{aligned} {\mathbb P}^{g}\left( \tau > t \mid X(s)=x\right) = \exp \left( -\int _s^t \sum _{\ell \in \mathcal {R}}\lambda _\ell ^{g}(u, x)\,\textrm{d}u\right) . \end{aligned}$$

Note that in (B.9), the convergence/divergence is at an exponential rate and therefore, by (B.9) and Assumption 5.4,

$$\begin{aligned} {\mathbb P}^{g}\left( \tau > t_k \mid X(s)=x\right) = 0 \end{aligned}$$

for all $s<t_k$ whenever $L_kx\ne v_k$. Hence, if $L_kX(s)\ne v_k$ for any $s<t_k$, *X* jumps before time $t_k$ with probability 1. Since this holds for any $s<t_k$, *X* jumps an infinite amount of times if $L_kX$ does not hit $v_k$.

We now consider the jumps that take $L_kX$ closer to $v_k$ with respect to the metric $d_k$. Let

$$\begin{aligned} \tau _+ = \inf \{t\ge s :d_k(v_k,L_kX(t))<d(v_k, L_kX(s))\}.\end{aligned}$$

Then the distribution of $\tau _+$ satisfies for any *x* such that $L_kx\ne v_k$ and $t_{k-1}<s<t<t_k$,

$$\begin{aligned} {\mathbb P}^{g}(\tau _+ > t\mid X(s)=x) \le \exp \left( -\int _s^t \sum _{\ell \in \mathcal {R}}\lambda _\ell ^{g}(u,x)\textbf{1}_{\{d_k(v_k, L_k(x+\xi _\ell ))<d_k(v_k,L_k x)\}} \,\textrm{d}u \right) .\end{aligned}$$

By Assumption 5.4, there are nonzero entries in this summation that tend to $\infty$ exponentially as $u\uparrow t_k$ and thus

$$\begin{aligned} {\mathbb P}^{g} \left( \tau _+>t_k\mid X(s)=x\right) =0 \end{aligned}$$

for any $s<t_k$ and $L_kx\ne v_k$. In other words, for any $s<t_k$ and $L_k x\ne v_k$,

$$\begin{aligned} {\mathbb P}^{g}\left( \exists t\in [s,t_k]\text { such that }d_k(v_k,L_k X(t))< d(v_k,L_k x) \mid X(s)=x\right) =1. \end{aligned}$$

Now ${\mathbb S}$ is a discrete lattice and thus any set ${\mathbb S}\cap K$, where $K\subseteq {\mathbb R}^d$ is compact, contains only finitely many points. Hence, since $L_k X$ keeps jumping with probability 1 to points strictly closer to $v_k$ if it does not hit $v_k$, it must hit $v_k$ with probability 1 and thus

$$\begin{aligned} {\mathbb P}^{g}\left( \exists t\in [s,t_k]\text { such that }L_kX(t)=v_k\mid X(s)=x\right) =1 \end{aligned}$$

for any *x* with $L_kx\ne v_k$. Since this obviously also holds for $L_kx=v_k$, we have by the law of total probability

B.10 $$\begin{aligned} {\mathbb P}^{g}\left( \exists t\in [s,t_k]\text { such that }L_kX(t)=v_k\right) =1. \end{aligned}$$

Now denote the collection of events $B_s = \bigcup _{s\le t\le t_k} \{L_kX(t)=v_k\}$, $s\in [t_{k-1},t_k]$. By (B.10), ${\mathbb P}^{g}(B_s)=1$ for all *s*. Since $B_s \downarrow \{L_kX(t_k)=v_k\}$ as $s\uparrow t_k$, we have by monotonicity of measures that ${\mathbb P}^{g}(L_kX(t_k)=v_k)=\lim _{s\uparrow t_k}{\mathbb P}^{g}(B_s)=1$. $\square$

## Appendix C Additional Lemmas

### Lemma C.1

Let *g* be defined by (4.7). Then for all *x*, *g*(*t*, *x*) is bounded and bounded away from zero uniformly in *t*.

### Proof

Note

$$\begin{aligned} (v-Lx)^{\prime }M(t)(v-Lx) \ge \lambda _{\min }(M(t))\left|v-Lx \right|^2 = \left( \lambda _{\max }(M^\dagger (t))\right) ^{-1}\left|v-Lx \right|^2.\end{aligned}$$

Since for all symmetric matrices *A*, *B*,

$$\begin{aligned}\lambda _{\min }(A)+\lambda _{\min }(B) \le \lambda _{\min }(A+B)\le \lambda _{\max }(A+B)\le \lambda _{\max }(A)+\lambda _{\max }(B),\end{aligned}$$

we have that

$$\begin{aligned} \left( \lambda _{\max }(M^\dagger (t))\right) ^{-1} \ge \left( \lambda _{\max }(C)+\lambda _{\max }(LaL^{\prime })(T-t)\right) ^{-1}.\end{aligned}$$

Now $LaL^{\prime }$ is positive definite and thus the right hand side is decreasing in *t*. Hence, a lower bound is given by $\left( \lambda _{\max }(C) + T\lambda _{\max }\left( LaL^{\prime }\right) \right) ^{-1}$. We conclude

$$\begin{aligned} g(t,x) \le \exp \left( -\frac{1}{2} \left|v-Lx \right|^2\left( \lambda _{\max }(C) + T\lambda _{\max }\left( LaL^{\prime }\right) \right) ^{-1} \right) . \end{aligned}$$

In a similar way, we can show

$$\begin{aligned} g(t,x) \ge \exp \left( -\frac{1}{2} \left|v-Lx \right|^2\lambda _{\min }(C)^{-1}\right) . \end{aligned}$$

$$\square $$

### Corollary C.2

(Multiple observations) Let *g* be defined by (4.14). Then for all *x*, *g*(*t*, *x*) is bounded and bounded away from zero uniformly in *t*.

### Proof

The proof of Lemma C.1 can be repeated using, for $t\in (t_{k-1},t_k]$,

$$\begin{aligned} \left|v(t)-L(t)x \right|^2 = \sum _{j=k}^n\left|v_j-L_jx \right|^2 \in \left[ \left|v_n-L_nx \right|^2, \sum _{j=1}^n \left|v_j-L_jx \right|^2 \right] .\end{aligned}$$

$$\square $$

## Appendix D Change of generator under change of measure

Here, we briefly recap the argument given in Section 4.1 of Palmowski and Rolski (2002). Fix *g* and define $\{\tilde{\mathbb {P}}_t,\, t\ge 0\}$ by

$$\begin{aligned} \frac{\,\textrm{d}\tilde{\mathbb {P}}_t}{\,\textrm{d}{\mathbb P}_t} = E^g(t):= \frac{g(t,X(t))}{g(0,x_0)} \exp \left( -\int _0^t \frac{\mathcal {A} g}{g}(s,X(s)) \,\textrm{d}s \right) ,\end{aligned}$$

where $\mathcal {A}$ is the generator of the space-time process under ${\mathbb P}$. We aim to show that under $\tilde{{\mathbb P}}$ the generator of this process is given by $\tilde{\mathcal {A}}$ where $g\tilde{\mathcal {A}}f= \mathcal {A}(fg)-f \mathcal {A}g$. For this, it suffices to prove that

$$\begin{aligned} \tilde{D}^f(t):= f(t,X(t)) - \int _0^t (\tilde{\mathcal {A}}f)(s, X(s)) \,\textrm{d}s \end{aligned}$$

is a local martingale under $\tilde{{\mathbb P}}$. To show this, by Lemma 3.1 in Palmowski and Rolski (2002) $\tilde{D}^f(t)$ is a local $\tilde{{\mathbb P}}$-martingale if and only if $\tilde{E}^f(t)$ is a local $\tilde{{\mathbb P}}$-martingale, where $\tilde{E}^f(t)$ is defined as $E^f(t)$ but with $\mathcal {A}$ replaced by $\tilde{\mathcal {A}}$. To show that $\tilde{E}^f(t)$ is a local $\tilde{{\mathbb P}}$-martingale, it suffices to show that $\tilde{E}^f(t) E^g(t)$ is a local ${\mathbb P}$-martingale (Cf. Lemma 4.1 in Palmowski and Rolski (2002)). Straightforward calculus gives

$$\begin{aligned} \tilde{E}^f(t) E^g(t) = E^{fg}(t), \end{aligned}$$

which is indeed a local ${\mathbb P}$ martingale.
